# Analysis of the tripartite interactions between two bacterial symbionts, a novel *Solitalea*-like bacterium (Bacteroidota) and *Cardinium*, and the stored product mite *Tyrophagus putrescentiae* based on gene expression data

**DOI:** 10.1128/spectrum.00609-25

**Published:** 2025-06-16

**Authors:** Jan Hubert, Qing Xiong, Eliza Glowska-Patyniak, Elizabeth V. Furtak, Pavel B. Klimov

**Affiliations:** 1 Department of Microbiology, Nutrition and Dietetics, Faculty of Agrobiology, Food and Natural Resources, Czech University of Life Sciences Prague 48371, Prague, Czechia; 2 School of Biomedical Sciences, The Chinese University of Hong Kong 130379, Hong Kong, China; 3 Department of Health Technology and Informatics, The Hong Kong Polytechnic University 539456, Hong Kong, China; 4 Department of Animal Morphology, Faculty of Biology, Adam Mickiewicz University in Poznan, Poznan, Poland; 5 Computer Science Department, University of Maryland 1068 , College Park, Maryland, USA; 6 Lilly Hall of Life Sciences, Purdue University 311308 , West Lafayette, Indiana, USA; Panepistemio Thessalias Tmema Geoponias Ichthyologias kai Ydatinou Periballontos, Volos, Greece

**Keywords:** Bacteroidota, Bacteroidetes, symbionts, *Cardinium*, mite, interaction, gene expression

## Abstract

The intracellular parasite *Cardinium* influences the bacterial microbiome composition of arthropod hosts; however, the mechanisms involved remain poorly understood. We sought to evaluate the interactions between *Cardinium* (cTPut) and SOL in *Tyrophagus putrescentiae* cultures based on relative abundance and gene expression data. First, we assembled the genome of *Candidatus* Krakonobacterium acarorum (formerly the *Soliltalea*-like symbiont SOL), a novel lineage of the Bacteroidota symbiont of mites. The assemblage SOL genome (1.2 Mb) contained complete pathways for the biosynthesis of lipoic acids, pantothenate, and menaquinone from futalosine. SOL is considered a facultative inhabitant (with prevalences ranging from 36% to 80% among individuals) of the gut (from 10^2^ to 10^4^ copies/mite) that is not detected in eggs, suggesting an extracellular location in the gut of mites. Second, gene expression was analyzed in SOL-inhabited cultures, including two cultures with cTPut and two cultures without cTPut. Correlation-based evidence for competition between cTPut and SOL was found mainly in the expression of transporter proteins. The presence of cTPut decreased interactions between SOL and the mite host; however, SOL is under greater control by mites in the presence of cTPut than in the absence of cTPut. Mite KEGG gene expression levels in the peroxisome, autophagy, sphingolipid, apoptosis, PI3K–Akt, and lysozyme pathways were more strongly correlated with SOL gene expression in cultures without cTPut than in those with cTPut. In contrast, mite KEGG gene expression levels in the proteasome, NF-κB, TNF, calcium, and Rap1 signaling pathways were more strongly correlated with SOL in the presence of cTPut. The explanation for these results is that cTPut mostly interacts with the mite host, resulting in changes in the host’s immunity-related/regulatory pathways, indirectly affecting the symbiont SOL.

**IMPORTANCE:**

Here, we describe the novel Bacteroidetes symbiont (SOL) of mites. The analysis of gene expression in meta-transcriptomic samples from cultures with and without the intracellular parasite *Cardinium* revealed the effect of *Cardinium* on SOL as a model facultative symbiont of mites. Our findings suggest that there is competition between these two symbionts for nutrients. In addition, *Cardinium* can influence other bacterial symbionts via mite host immunity-related and regulatory pathways. *Tyrophagus putrescentiae* is a cosmopolitan pest mite that contaminates the home environment, including stored food and feed, with allergens. The interactions between intracellular bacteria and other members of the microbiome influence host physiology and indirectly affect allergen production.

## INTRODUCTION

Many arthropods harbor several species of bacterial symbionts within the same host individual (1, 2). Mutualistic bacteria can influence host adaptation to a habitat by (i) providing essential nutrients such as amino acids, fatty acids, and vitamins in situations with limited nutrition (3) and (ii) increasing host resistance to external biotic or abiotic stress factors, including pathogens, parasitoid attacks, temperature changes, and insecticides (3, 4). Reproductive parasites are among these bacteria, and they influence host growth, reproduction, and survival to spread in host populations (5, 6). For example, *Cardinium* is a maternally transmitted intracellular symbiont that is widely distributed among arthropods (7) and has been reported in 45 species of mites (8). To date, 10 *Cardinium* genomes have been described (9). *Cardinium* manipulates host feminization, parthenogenesis, and cytoplasmic incompatibility (CI) (10, 11). Moreover, *Cardinium* was found to be important in shaping the microbiome of *Tetranychus* species (12) and reducing bacterial diversity in the planthopper (*Nilaparvata lugens*) (13). However, little is known about how *Cardinium* affects other members of the host microbiome.

Based on 16S DNA amplicon sequencing, domestic mites from the Astigmata group are colonized by a wide spectrum of bacteria, such as the intracellular parasites *Cardinium* and *Wolbachia,* facultative symbionts (*Bartonella*-like symbionts, *Blattabacterium*-like symbionts, *Erwiniaceae* symbionts, and a *Solitalea-*like symbiont [SOL]) that occur in the gut and are present in the fecal fraction (14–16). Based on the microbiome profile from V1 to V3 16S DNA sequencing of various *Tyrophagus putrescentiae* cultures, we observed two separate co-occurring groups of bacteria: *Wolbachia* co-occurs with *Blattabacterium*-like symbionts, *Bartonella*-like symbionts, and *Solitalea*-like symbionts, whereas *Cardinium* is distinctly separated from these symbionts and has a negatively correlated profile (17, 18).

Through phylogenetic analyses of the 16S rRNA gene, in the stored-product mites *Acarus siro* and *T. putrescentiae*, SOL belongs to the phylum Bacteroidota and forms separate lineages (19). The relative abundances of SOL OTU_97_ (operational taxonomic unit at 97% sequence similarity) were 95% in the *A. siro* microbiome and 25% in the *T. putrescentiae* microbiome (18, 20). SOL cells are located in fat tissues, reproductive organs, and the digestive tract of *A. siro* (19). Because *A. siro* cultures lack *Cardinium* (18), analyses of interactions are not possible. *T. putrescentiae* cultures present two scenarios, that is, SOL-inhabited cultures with and without cTPut (18–20). This raises the question of whether they interact with each other. These cultures also enable comparisons of the effects of cTPut to those of SOL.

Here, we compared the gene expression patterns between two cTPut-positive and two cTPut-negative hosts inhabited by SOL, which represent different cultures of *T. putrescentiae*, using previously obtained meta-transcriptome samples (14). Prior to these analyses, three novel genomic assemblies of SOL from metagenome samples of *T. putrescentiae* and *A. siro* were generated. Then, the relationships among SOL, cTPut, and their host *T. putrescentiae* were investigated using gene expression data: (i) the gene expression levels of SOL and cTPut were correlated in coinfected cultures; and (ii) the gene expression levels in SOL and the mite host were compared in cTPut-positive and cTPut-negative mite cultures.

## RESULTS

### Prevalence of SOL in mite cultures

Single-mite PCR revealed that among infected individuals, the prevalence of SOL ranged from 36% to 80%. qPCR with specific primers detected SOL in samples from mite bodies and spent growth medium (SPGM; fecal fraction) but not eggs from *T. putrescentiae* or *A. siro* cultures (Fig. S1). For all the tested surface-sterilized eggs, the SOL copy number was below the detection threshold (10 copies/egg) (Table S1). In mite bodies, the SOL copy number was tenfold greater in *A. siro* (approximately 10⁴ copies/mite) than in *T. putrescentiae* (ranging from 10² to 10³ copies/mite) (*T*_(1,41)_ = 10.26, *P* < 0.001). Higher copy numbers were detected in the SPGM (feces fraction in the mite rearing culture) of *A. siro* than in that of *T. putrescentiae* (*T*_(1,41)_ = 3.735, *P* < 0.001), although the SOL copy numbers exhibited high variability in the SPGM of *T. putrescentiae*. There was a difference between *T. putrescentiae* cultures in terms of the number of copies per mite (*T*_(1,10)_ = 4.050, *P* = 0.003). The number of cTPut copies per mite was approximately 10^2^. The number in culture 5L was slightly greater than that in culture 5S, with average log values as follows: 5L = 2.43 and 5S = 2.17.

### SOL genomes reveal a novel lineage of Bacteroidota, *Candidatus* Krakonobacterium acarorum

**Fig 1:**
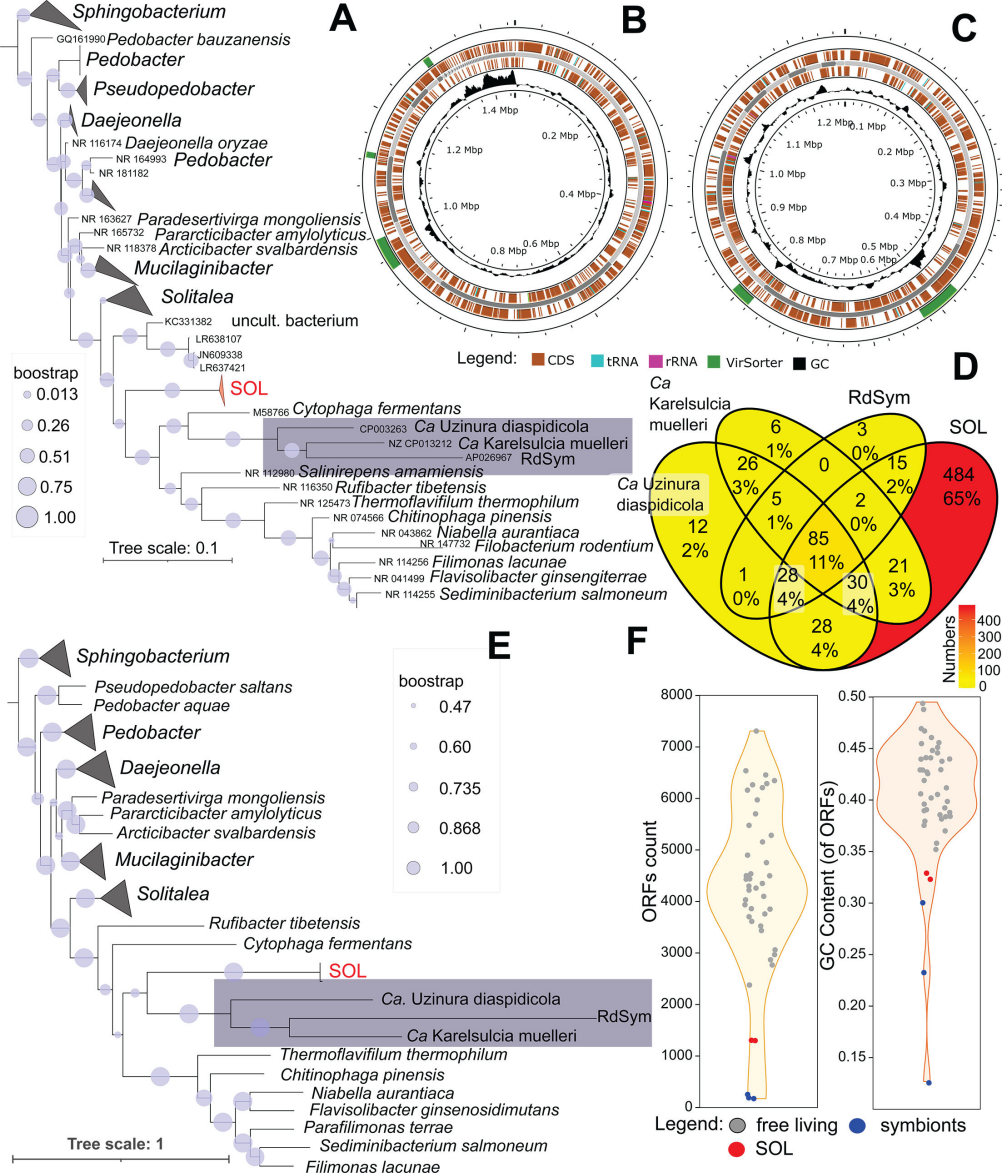
Phylogenetic analyses and genomic overview of the *Solitalea*-like symbiont (SOL). (**A**) Phylogenetic tree based on 16S rDNA (for input sequence data, see Table S4). The sequences were aligned and bootstrapped in arb-silva (GTR + R); the tree was rooted to *Olivibacter sitiensis* (NR_043805). (**B and C**) Coding regions of the genomes visualized in PROKSEE. (**B**) SOL from *Tyrophagus putrescentiae*. (**C**) SOL from *Acarus siro*. (**D**) Venn diagram comparison of the predicted proteins annotated in KEGG (Table S6) among Bacteroidetes symbionts. (**E and F**) Comparison of the genomes in M1CR0B1AL1Z3R (24) (for input sequences, see Table S5). (**E**) Phylogenetic tree rooted to *Olivibacter sitiensis* (NZ_ATZA01000001). (**F**) Violin and jitter plot comparisons of the numbers of ORF counts and GC contents of ORFs. The SOL symbiont is indicated in red, and the next symbiont is indicated in blue.

Two SOL genomes were obtained from *T. putrescentiae* (Czech and Chinese cultures), and one was obtained from *A. siro* (Table 1; Fig. 1). The SOL genomes from *T. putrescentiae* and *A. siro* presented 95% and 90% completeness, respectively, as assessed using CheckM on KBase for Bacteroidota (=Bacteroidetes) (21, 22). The three genomes shared 99.3–99.9% average nucleotide identity (23) (Fig. S2). The SOL genomes from *T. putrescentiae* encoded 1138 and 1389 CDSs and 724 and 790 KEGG proteins (Table 1) for the Chinese and Czech mite cultures, respectively (Fig. 1; Table S2 and S3).

**TABLE 1:** Assembly statistics for three genomes of the new *Solitalea-like* symbiont (SOL) from the stored-product mites *Tyrophagus putrescentiae* (Czechia and China) and *Acarus sir*o (Czechia) calculated in checkM^*a*^^,*b*^

Genome	NCBI	Size	Compl%	Cont%	Contigs	GC %	CDSs	rRNA	tRNA	KEGG	Coverage
SOL_Tyr_put_Chinese	JBNOKO000000000	1,312,108	95	0.48	1	31.8	1,138	6	35	724	23
SOL_Tyr_put_CZ	JBNOKP000000000	1,598,319	95	0.48	117	32.7	1,273	3	33	790	1,252
SOL_Aca_sir	JBNOKQ000000000	1,275,755	90	0	11	31.6	1,278	3	32	862	9

^*a*^ Compl.—completeness; Cont. —contamination. The Czech genome was assembled from different *Tyrophagus putrescentiae* cultures, whereas the Chinese genome was assembled from a single culture. The genomes were submitted project PRJNA1196338. The coverage was calculated based on metagenome reads.

^*b*^ KEGG proteins were identified in GhostKOALA. Mite culture data are provided in Table S1 and S6.

SOL is a new species and probably a representative of a new group of symbionts, as no similar genomes were found using the Type Strain Genome Server (25, 26) (Fig. S3 and S4). We suggest naming this bacterial genus (SOL) *Candidatus* Krakonobacterium acarorum (from Krakonos, a Bohemian folkloric mountain spirit [woodwose] of the Giant Mountains (Krkonose Mts., Karkonosze Mts.) and bacterium; acarorum refers to the host associations of this new bacterium). BLAST comparison of the 16S RNA gene revealed 99.86–100% similarity to SOL sequences from our previous studies (20, 27). The 16S RNA analyses confirmed earlier findings. The 16S rDNA of SOL also shared 85% similarity with those of *Solitalea canadensis* (NR_074099), *Pseudopedobacter saltan* (NR_074586), and *Pedobacter gandavensis* (NR_181180). The new SOL cluster was also supported by our analysis of 16S rDNA sequences from GenBank (Table S4), where SOL formed a separate group outside *Solitalea* (Fig. 1). A genomic comparison of M1CR0B1AL1Z3R (24) revealed some similarity between SOL and other Bacteroidetes symbionts, that is, the scale insect (Coccoidea: Hemiptera) symbiont *Ca.* Uzinura diaspidicola (28), the sap-feeding insect symbiont *Ca.* Karelsulcia muelleri (29) and the symbiont *Rhyzopertha dominica* (RdSym) (30, 31) (Fig. 1). However, all these symbionts had smaller genomes, which featured fewer open reading frames (ORFs). The GC content of the ORFs decreased in the following order: free-living bacteria > SOL > other Bacteroidetes symbionts (Fig. 1). A comparison of the predicted KEGG proteins among SOL and other Bacteroidetes symbionts revealed that most proteins were unique to SOL (65%), and only 11% of the predicted proteins were shared by all symbionts (Fig. 1). MASH average nucleotide analyses using the selected genomes of Sphingobacteriales (Table S5) placed SOL together with other Bacteroidetes symbionts as a sister group of free-living *Solitalea* (Fig. S5), further indicating that SOL belongs to a novel lineage. Based on V4 16S DNA comparison, SOL was distinct from ant symbionts, which also formed a new genus of the order Sphingobacteriales (32, 33) (Fig. S6).

The genome of SOL includes 26 complete KEGG pathway modules, and analysis of these modules indicated that SOL could synthesize lipoic acid, pantothenate, and menaquinone from futalosine. The nucleotide metabolism pathways include purine and pyrimidine biosynthesis, adenine and guanine biosynthesis, and proline degradation. SOL has a Sec-SRP secretion system, although SecM and SecB were not identified. Instead, the secretin protein GspD, the outer membrane protein TolC, and the twin-arginine-targeting proteins TatA and TatC were identified (Table S2). The ABC transporters include proteins for phospholipid transport (MlaDEF), lipoproteins (LolCED), lipopolysaccharide (LptBFG), and heme (CcmBC) transport. Other proteins with transport functions include FtsX, FtsE, and MsbA (Table S2).

### SOL gene expression in cTPut_positive and cTPut_negative cultures of *T. putrescentiae*

**Fig 2:**
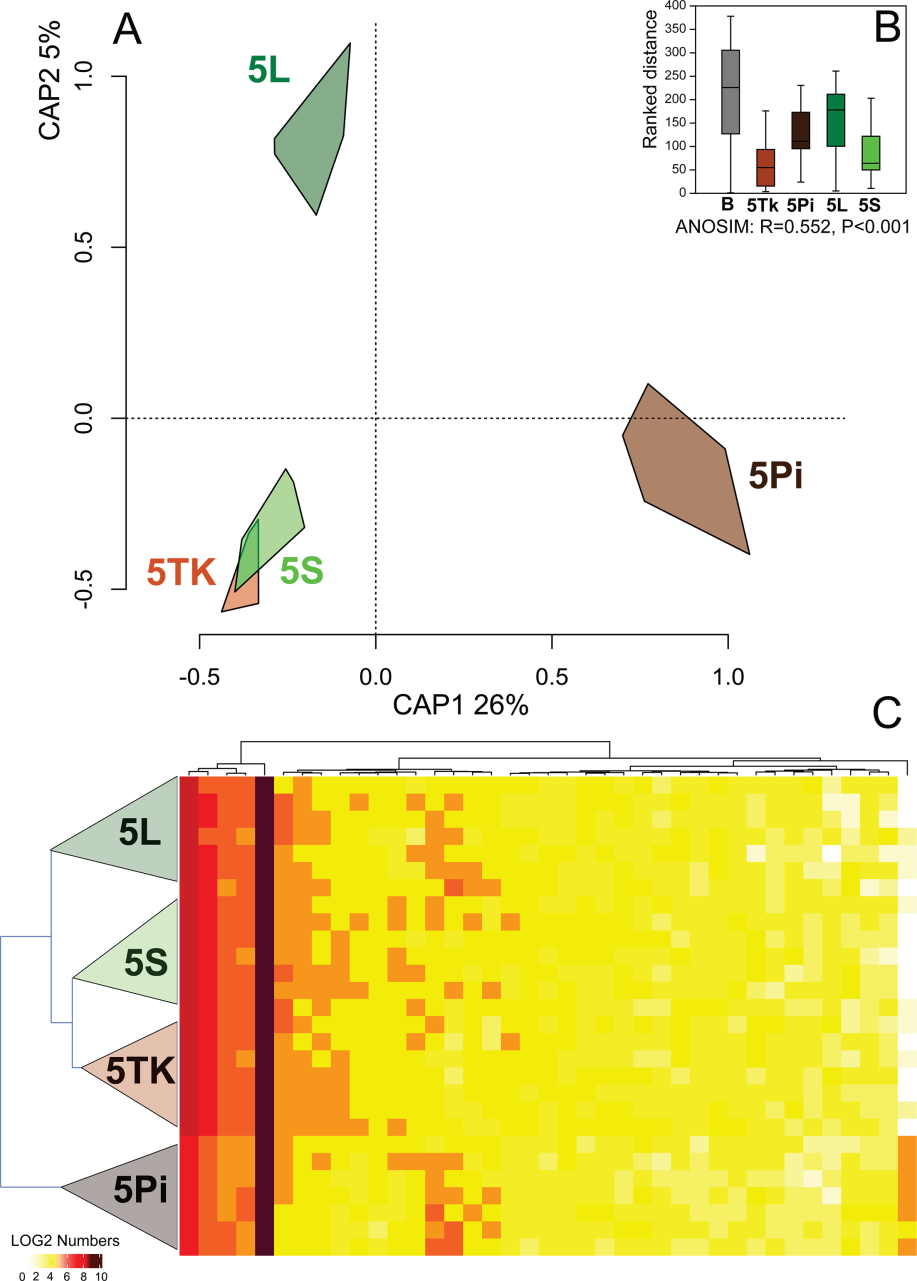
SOL gene expression in *Tyrophagus putrescentiae* samples with and without cTPut. (**A**) Ordination plot from distance-based redundancy analysis (dbRDA) showing gene expression across mite cultures; samples from the same culture are visualized as convex hulls. (**B**) ANOSIM comparison of gene expression among mite cultures. (**C**) Heatmap showing the expression profiles of the most abundant genes (log2-transformed). B = all cultures; 5L, 5S = cTPut-positive cultures; 5Tk, 5Pi = cTPut-negative cultures.

The relative abundance of SOL to mite reads was significantly greater in samples without cTPut than in samples with cTPut (Mann–Whitney *U*-test = 21, *z* = 3.315, *P* < 0.001) (Fig. S7A). The presence of cTPut explained 8% of the variance in SOL gene expression (distance-based redundancy analysis [dbRDA]: F_(1,26)_=2.567, *P* = 0.005). Similar results were obtained via ANOSIM (*R* = 0.140, *P* < 0.008). However, a dbRDA with additional predictors demonstrated that the mite culture variable explained 34% of the variance in SOL gene expression (*F*_(3,24)_ = 4.101, *P* < 0.001), whereas the effect of cTPut was not significant. An ordination analysis revealed similarities between *Cardinium*-positive samples along the CAP1 axis; however, a combination of the CAP1 and CAP2 axes grouped two disparate samples: 5S (cTPut-positive) and 5TK (cTPut-negative) (Fig. 2A). When SOL gene expression was compared using false discovery rate (FDR) analysis, only five genes showed significant differences (Table S7), which may be an artifact of their low abundance. Similarly, no significant differences in gene expression were detected between cTPut-positive and cTPut-negative cultures based on the Shannon diversity index (Mann–Whitney *U*-test = 71, *z* = 1.212, *P* = 0.233) (Fig. S7B; Table S8).

The most highly expressed SOL genes were those involved in replication (cell division protein FtsQ) and transport (transporters msbA and ftsX), followed by glutaminyl-peptide cyclotransferase QCT, CcmD family protein, and unidentified proteins (LOCUS_12640) (Table S2). Overall, our results indicate that SOL was significantly more abundant in cTPut-negative samples than in control samples, but its gene expression did not vary with the presence or absence of cTPut.

### Correlations between SOL and cTPut gene expression in the *T. putrescentiae* metatranscriptome

**Fig 3:**
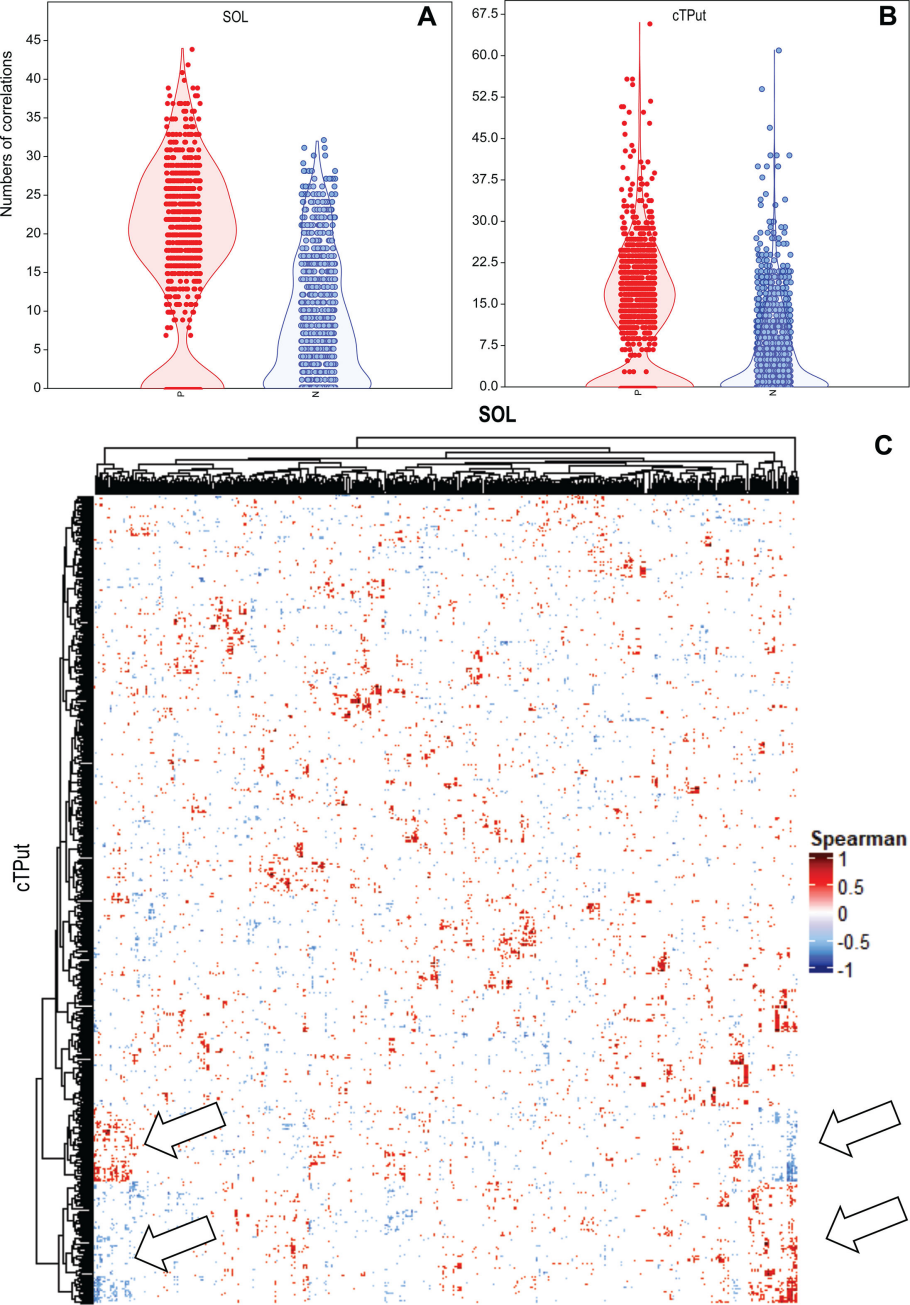
Correlation of cTPut and SOL gene expression in cultures 5L and 5S. Spearman correlations (permutation-based *P* < 0.05) were calculated. (**A and B**) Violin and jitter plots showing the numbers of positive (red) and negative (blue) correlations per gene. (**A**) SOL and (**B**) cTPut. (**C**) Correlation matrix clustered by the Ward method, with arrows pointing to gene clusters with the highest numbers of correlations.

Two dbRDA analyses, with SOL gene expression as the dependent variable and cTPut gene expression (Tables S9 and S10) as the independent variable and vice versa, yielded very similar results. Both models explained nearly the same amount of variance (31%), indicating a moderate level of association (Table 2, Model 3: *R* = 0.311; Model 4: *R* = 0.316). The gene expression profiles of both symbionts showed 15,753 positive and 7,819 negative correlations in cultures 5L and 5S, respectively (Fig. 3). Four clusters with potential regulatory functions between cTPut and SOL were identified (Fig. 3).

**TABLE 2:** Distance-based redundancy analysis (dbRDA) of the correlations between SOL and cTPut gene expression and host mite KEGG gene expression^*a*^^,^^*b*^^,^^*c*^

Nr	Dependent	Factor	With cTPut				Without cTPut			
			df		*R*	*F*	df		*R*	*F*
			Mod.	Res.			Mod.	Res.		
1	Mite KEGG	SOL	2	11	0.203	1.403	2	11	0.203	1.402
2	SOL	Mite KEGG	3	10	0.327	1.618	6	7	0.643	2.101
3	SOL	cTPut	3	10	0.311	1.508				
4	cTPut	SOL	3	10	0.316	1.542				
5	Mite KEGG immune	SOL	2	11	0.219	1.540	3	10	0.307	1.474
6	SOL	Mite KEGG immune	3	10	0.326	1.611	5	8	0.537	1.854
7	Mite_metabolism	SOL	6	7	0.919	13.268	7	6	0.947	15.343
8	SOL	Mite metabolism	2	11	0.218	1.536	3	10	0.316	1.542

^*a*^ Immune—Mite KEGG genes involved in immune and regulatory pathways (*n* = 1057); df—Degrees of freedom for the model (mod.) and residuals (res.); F—*F* value from the Monte Carlo permutation test. All tests were significant at *P* < 0.05. For models 3 and 4, the situation without cTPut was not calculated because cTPut was used as an environmental variable.

^*b*^ Partial models were calculated for cultures with (5L and 5S) and without cTPut (5Pi and 5Tk). Different combinations of dependent and independent variables were used, with model parameters shown. The Bray–Curtis distances of the models were calculated.

^*c*^ Empty cells indicate not observed.

Gene expression correlation network analysis (Table S11) via Cytoscape identified 21 cTPut genes, most of which were associated with genetic information processing (50%) and membranes (e.g., the hypothetical proteins GenBank WP_260536282 and WP_260537419). Among the 34 SOL genes, the expression of two ABC membrane transporters (Locus_12380 and ftsX) was positively correlated with cTPut gene expression (Fig. S8).

### Correlations between SOL gene expression and *T. putrescentiae* KEGG gene expression in samples with and without *Cardinium*

dbRDA models were constructed for *T. putrescentiae* cultures with (5S and 5L) and without cTPut (5Pi and 5Tk). The expression of the mite KEGG genes (Table S12) was only marginally influenced by SOL gene expression, as indicated by the low explained variance (Table 2, model 1: *R* = 0.203). However, when SOL gene expression was tested as a dependent variable, and mite KEGG gene expression was tested as an independent variable, the models provided different results. SOL gene expression, influenced by mite KEGG genes, had a slightly stronger effect in the presence of cTPut (*R* = 0.327) and a much stronger effect in its absence (*R* = 0.643).

Gene expression correlation networks (Fig. S9 and S10) revealed that the number of correlations per mite gene with SOL was lower in cultures containing cTPut than in those without cTPut (mean = 25 vs 21; *t*-test: positive *t* = −21.78, negative *t* = −29.838, *P* < 0.05). A similar trend was observed in the analysis of correlations per SOL gene (positive *t* = −4.188, negative *t* = −5.3741, *P* < 0.05) (Fig. S11). In total, there were 122,558 positive and 107,110 negative correlations in the cultures with cTPut, whereas 147,145 positive and 139,568 negative correlations were detected in the samples without cTPut. Overall, our results suggest that the presence of cTPut decreased the interaction between SOL and the mite host.

### Correlations between the expression of SOL genes and *T. putrescentiae* immunity-related and regulatory KEGG genes in samples with and without *Cardinium*

Overall, the results of our dbRDA analysis of the gene expression patterns of both mites and SOL reflected the trends described above (see Table 2, models 5 and 6, and models 1 and 2 for comparisons). Interactions between SOL and mite immunity-related and KEGG regulatory pathways differed between samples with and without cTPut, with 11 pathways showing differences (Table 3, terms in bold). The correlations between KEGG gene expression in the peroxisome, autophagy, sphingolipid, apoptosis, PI3K–Akt, and lysozyme pathways and SOL gene expression were greater in cultures with cTPut than in those without cTPut (Table 3). In contrast, KEGG gene expression profiles in the proteasome, NF-kappa B, TNF, calcium signaling, and Rap1 signaling pathways were strongly correlated with SOL gene expression in cultures without cTPut (Table 3).

**TABLE 3:** Distance-based redundancy analysis (dbRDA) showing correlations between predicted mite KEGG gene expression in immune and regulatory pathways and SOL expression^*a*^^,^^*b*^

Nr		With cTPut				Without cTPut				dR
		df		*R*	*F*	df		*R*	*F*	
		Mod.	Res.			Mod.	Res.			
**1**	**Peroxisome^*c*^**	8	5	0.837	3.214	5	8	0.623	2.640	0.215
**2**	**Autophagy**	6	7	0.778	4.090	5	8	0.654	3.021	0.124
**3**	**Sphingolipid**	10	3	0.962	7.675	7	6	0.852	4.947	0.110
**4**	**Apoptosis**	9	4	0.908	4.389	7	6	0.825	4.045	0.083
**5**	**PI3K_Akt**	6	7	0.667	2.334	5	8	0.585	2.252	0.082
**6**	**Lysozyme**	7	6	0.860	5.282	6	7	0.782	4.187	0.078
7	FoxO	6	7	0.728	3.129	5	8	0.674	3.300	0.055
8	TGF_beta	8	5	0.909	6.226	7	6	0.859	5.231	0.050
9	RAS	7	6	0.772	2.894	6	7	0.732	3.190	0.039
10	Ubiquitin	5	8	0.654	3.017	5	8	0.615	2.559	0.038
11	TOLL_IMD	7	6	0.866	5.543	7	6	0.834	4.290	0.033
12	Wnt	8	5	0.835	3.166	7	6	0.804	3.518	0.031
13	ErbB	8	5	0.858	3.773	7	6	0.835	4.324	0.023
14	AMPK	7	6	0.809	3.629	6	7	0.792	4.434	0.017
15	Insulin	7	6	0.838	4.430	6	7	0.822	5.374	0.016
16	Bact_invasion of EC	7	6	0.808	3.613	6	7	0.795	4.530	0.013
17	Hedgehog	8	5	0.867	4.061	7	6	0.855	5.056	0.012
18	Phosphatidylinositol	6	7	0.828	5.602	7	6	0.828	4.111	0.000
19	Phospholipase_D	6	7	0.820	3.900	7	6	0.827	4.094	−0.007
20	Mitophagy	6	7	0.748	3.457	6	7	0.757	3.625	−0.009
21	TOLL	8	5	0.899	5.573	8	5	0.911	6.370	−0.011
22	Phagosome	7	6	0.902	7.863	8	5	0.915	6.702	−0.013
23	Notch	7	6	0.888	6.775	8	5	0.910	6.298	−0.022
24	Apelin	7	6	0.824	4.018	7	6	0.851	4.887	−0.027
25	Reg._actin_cytoskel.	6	7	0.712	2.883	6	7	0.743	3.366	−0.031
26	JAK_STAK	8	5	0.891	5.118	8	5	0.923	7.469	−0.032
27	cAMP	6	7	0.655	2.214	6	7	0.697	2.689	−0.043
28	NOD	6	7	0.787	4.298	7	6	0.830	4.186	−0.044
29	HIF_1	6	7	0.840	6.111	6	7	0.886	6.670	−0.046
30	mTOR	6	7	0.707	2.808	7	6	0.755	2.638	−0.048
31	p53	6	7	0.738	3.284	7	6	0.793	3.278	−0.055
32	Endocytosis	5	8	0.580	2.211	6	7	0.645	2.121	−0.065
33	MAPK	6	7	0.692	2.621	7	6	0.761	2.728	−0.069
34	Hippo	6	7	0.730	3.158	7	6	0.801	3.450	−0.071
35	Oocyte meiosis	6	7	0.715	2.923	7	6	0.787	3.168	−0.072
36	cGMP_PKG	6	7	0.779	4.104	7	6	0.852	4.935	−0.073
37	**Protesome**	6	7	0.782	4.189	7	6	0.862	5.333	−0.079
38	**NF_kappa_B**	7	6	0.808	3.611	8	5	0.900	5.634	−0.092
39	**TNF**	6	7	0.749	3.489	7	6	0.843	4.612	−0.094
40	**Calcium_signaling**	5	8	0.631	2.740	6	7	0.740	3.315	−0.108
41	**Rap1**	6	7	0.699	2.712	8	5	0.867	4.056	−0.167

^*a*^ Immune—Selected mite KEGG genes involved in mite immune and regulatory pathways; df—Degrees of freedom explained by the model (mod.) and residual (res.); F—*F* value of the Monte Carlo permutational test. All tests were significant at *P* < 0.05; R—Variability explained by the model; dR—R (cTPut-positive model) minus R (cTPut-negative model). In these models, SOL expression was used as the independent variable, and mite KEGG gene expression served as the dependent variable. Model parameters are provided. The data were calculated with a robust Aitchison distance.

^*b*^ Partial models were calculated separately for cultures with cTPut (5L and 5S) and without cTPut (5Pi and 5Tk).

^*c*^ Bold indicates pathways that differ (dR) between cTPut-positive and cTPut-negative samples.

The mite KEGG genes in these pathways were further analyzed via Cytoscape (Table S11). The analysis identified 29 mite genes and 28 SOL genes with the highest numbers of correlations (Tables 4 and 5). The mite genes interacted with the SOL membrane proteins OmpA, citrate synthase, and glutaminyl-peptide cyclotransferase and a hypothetical protein in cTPut-positive cultures. In contrast, the Type II toxin-antitoxin system antitoxin, RelB/DinJ family, deoxyhypusine synthase, and excinuclease ABC genes presented the strongest correlations with mite immune and regulatory genes in cTPut-negative cultures.

**TABLE 4:** SOL genes identified by Cytoscape correlation network analysis of gene expression among symbiont and mite immunity-related and regulatory pathway KEGG genes in *cTPut*-positive and *cTPut*-negative samples^*a*^^,^^*b*^

*Solitalea*-like	Name	Description	KEGG	cTPut-negative			cTPut-positive		
				*N*	S_type	S_strength	*N*	S_type	S_strength
LOCUS_00570	fabD	ACP S-malonyltransferase	K00645	13	13	65	6	−4	58
LOCUS_00800	OmpA	Outer membrane protein OmpA		27	21	182			
LOCUS_00920		Hypothetical protein		8	2	60	9	−9	59
LOCUS_01380	groL	Chaperonin GroEL	K04077	25	19	166	1	1	2
LOCUS_01450	ftsX	Permease-like cell division protein FtsX	K09811	17	−9	128	4	2	19
LOCUS_01580	uvrB	Excinuclease ABC subunit UvrB	K03702	5	−5	26	44	−2	251
LOCUS_02420	RP-S16	30S ribosomal protein S16	K02959	10	−8	39	1	1	1
LOCUS_02770		Hypothetical protein		18	10	107			
LOCUS_03410		BZIP transcription factor		26	16	137			
LOCUS_03680	TC.OOP	OmpA-OmpF porin, OOP family	K03286	26	12	125	2	−2	2
LOCUS_04290	mfd	Transcription-repair coupling factor	K03723	17	−7	65	36	10	238
LOCUS_04730	QCT	Glutaminyl-peptide cyclotransferase	K22757	35	13	197	9	−3	57
LOCUS_04780	pheS	Phenylalanine-tRNA ligase subunit alpha	K01889	3	1	8	33	−33	197
LOCUS_04810		Hypothetical protein		30	10	223			
LOCUS_05310	DHPS	Deoxyhypusine synthase family protein	K00809	2	2	16	59	−41	366
LOCUS_05560	ubiD	4-hydroxy-3-polyprenylbenzoate decarboxylase [EC:4.1.1.98]	K03182				30	6	235
LOCUS_05850		Hypothetical protein	K06133	20	−18	148	4	−4	23
LOCUS_06680		Outer membrane protein beta-barrel domain-containing protein		25	−15	120			
LOCUS_07120	REV3L	DNA polymerase zeta [EC:2.7.7.7]	K02350	24	18	148	1	−1	4
LOCUS_07200		Citrate synthase	K01647	27	15	204	2	−2	20
LOCUS_09730		Type II toxin-antitoxin system antitoxin, RelB/DinJ family		1	1	10	30	−24	236
LOCUS_10120	secDF	Protein translocase subunit SecDF	K12257	27	15	177	1	1	10
LOCUS_10190		Dihydroorotase	K01465	12	−10	60	1	−1	4
LOCUS_10410	recR	Recombination mediator RecR	K06187				51	43	302
LOCUS_11430		Hypothetical protein					25	−3	140
LOCUS_11500		Hypothetical protein		6	0	38	15	11	77
LOCUS_11610		Hypothetical protein		21	15	114	3	1	26
LOCUS_12490		ATP-binding cassette, subfamily B, bacterial MsbA	K11085	17	−13	102			

^*a*^ N—Number of KEGG genes in correlation; S_type—Sum of the vector describing positive (1) and negative (−1) correlations to mite KEGG genes; S_Strength—Sum of strength in the correlation. The most important genes are marked in gray, with value levels represented by a color gradient. This analysis used absolute Spearman correlation values between 0.75 and 1 for gene expression, permutational *P* < 0.05, and a total sum of strength > 100.

^*b*^ Empty cells indicate not observed.

**TABLE 5:** Mite KEGG genes identified by Cytoscape correlation network analysis of gene expression among symbiont and mite immune and regulatory pathway KEGG genes in *cTPut*-positive and *cTPut*-negative samples^*a*^^,^^*b*^

KEGG	Name	Description	cTPut-negative			cTPut-positive		
			*N*	S_type	S_strength	*N*	S_type	S_strength
K00922	PIK3CA_B_D	Phosphatidylinositol-4,5-bisphosphate 3-kinase	48	−12	195	24	−12	146
K01158	DNASE2	Deoxyribonuclease II	24	10	104			
K01201	GBA	Glucosylceramidase	30	14	143	2	−2	8
K01204	NAGA	Alpha-N-acetylgalactosaminidase	20	12	80	7	1	44
K01363	CTSB	Cathepsin B	40	20	185	4	0	15
K01379	CTSD	Cathepsin D	69	33	399	6	0	46
K02161	BCL2	Apoptosis regulator Bcl-2	18	−12	101	9	3	28
K02677	PRKCA	Classical protein kinase C alpha type	8	0	64	8	0	41
K03158	TNFRSF1A	Tumor necrosis factor receptor superfamily member 1A	4	−4	24	32	32	215
K04361	EGFR	Epidermal growth factor receptor	6	0	25	18	0	110
K04363	PDGFRA	Platelet-derived growth factor receptor alpha				18	18	174
K04368	MAP2K1	Mitogen-activated protein kinase kinase 1	24	24	148	18	−6	140
K04371	ERK	Mitogen-activated protein kinase 1/3	6	−6	34	30	−6	158
K04382	PPP2C	Serine/threonine-protein phosphatase 2A				15	−3	106
K04426	MAP3K5	Mitogen-activated protein kinase	45	−33	257	6	0	35
K04456	AKT	RAC serine/threonine-protein kinase	36	−12	288	6	6	24
K04465	NR4A1	Nuclear receptor subfamily 4 group A/1	24	−16	160	1	1	1
K04467	IKBKA	Inhibitor of nuclear factor kappa-B kinase	12	−4	75	12	−12	79
K05087	IGF1R	Insulin-like growth factor 1 receptor	6	0	22	12	12	92
K05209	GRIN2A	Glutamate receptor ionotropic, NMDA 2A	44	32	235			
K05692	ACTB_G1	Actin beta/gamma 1	20	10	104	2	−2	8
K06276	PDPK1	3-phosphoinositide dependent protein kinase-1	15	15	74	18	6	119
K08048	ADCY8	Adenylate cyclase 8	50	26	294	6	−2	54
K08601	CYLD	Ubiquitin carboxyl-terminal hydrolase CYLD	12	−6	92	6	0	46
K12386	CTNS	Cystinosin	24	10	91	4	0	17
K13239	ECI2	Delta3-Delta2-enoyl-CoA isomerase	22	8	143			
K13352	PEX11B	Peroxin-11B	14	−2	83	4	0	25
K16172	IRS1	Insulin receptor substrate 1	56	−28	353	6	6	38
K19662	PRKCB	Classical protein kinase C beta type	12	12	40	28	4	147

^*a*^ N—Number of KEGG genes in correlation; S_type—Sum of the vector describing positive (1) and negative (−1) correlations to mite KEGG genes; S_Strength—Sum of strength in the correlation. The most important genes are marked in gray, with value levels represented by a color gradient. This analysis used absolute Spearman correlation values between 0.75 and 1 for gene expression, permutational *P* < 0.05, and a total sum of strength > 100.

^*b*^ Empty cells indicate not observed.

The mite KEGG genes in immunity-related and regulatory pathways exhibited different interactions with SOL genes in cultures with and without cTPut (Table S12). The most notable genes were cathepsins B and D, glutamate receptor, adenylate cyclase, and insulin receptor substrate, which exhibited a high number of interactions with SOL genes in cTPut-positive cultures (Table S13). In contrast, tumor necrosis factor and platelet-derived growth factor receptor alpha strongly interacted with SOL genes in cTPut-negative samples (Fig. S12).

### Correlations between SOL genes and *T. putrescentiae* metabolic pathways in samples with and without *Cardinium*

The expression profiles of mite genes involved in metabolism were strongly influenced by SOL when tested as environmental variables, with this influence being stronger in cTPut-negative samples (Table 2, model 7: *R* = 0.947 vs *R* = 0.919). In contrast, the influence of mite metabolism, which is an environmental variable, on SOL was lower. Mite metabolism was a more important factor in the samples without cTPut than in the samples with cTPut (Table 2, model 8, *R* = 0.316 vs *R* = 0.281). These findings indicate that cTPut suppresses interactions between SOL and mite metabolism. FDR analyses revealed differences in 10 pathways between samples with and without cTPut, but the absolute values of the log_2_-fold changes were lower than 1, indicating that the differences in gene expression were low (Table S14).

## DISCUSSION

### *Candidatus* Krakonobacterium acarorum, a novel lineage of Bacteroidota symbiont of *A. siro* and *T. putrescentiae*

SOL is associated with major stored-product mites, such as *T. putrescentiae* in Europe (this study) and China (this study) (34) and *A. siro* in Europe, but likely has a broader global distribution and host range. We suggest that the SOL is horizontally transferred. Its absence in the eggs of the mites indicated that the qPCR results were below the detection thresholds. The same analyses confirmed the presence of SOL copies in mite bodies and feces, supporting previous results of amplicon sequencing of 16S DNA (18). The possible method of transfer is via the presence of SOL on the egg surface; the mites deposit eggs and feces, and cross-contamination of feces and egg surfaces is highly possible. In an infected culture, SOL is not present in all individual mites and likely represents a facultative symbiont (35). Its abundance varies across different phases of laboratory culture (19). The infection rates among individual mites range from 36% to 80%, and the SOL 16S DNA copy number ranges from 10² to 10⁴ per mite.

We characterized a new symbiont of stored-product mites, SOL belonging to the phylum Bacteroidota, using genomic and transcriptomic data. Our phylogenetic analysis revealed that SOL represents a completely new group of symbionts related to free-living *Solitalea.* However, SOL is distinct from the Sphingobacteriales symbiont recently described in ants (32, 33). As a characteristic trend of symbionts (36), genome reduction was observed in the SOL genome (1.3–1.6 Mb), which is smaller than that of free-living *Solitalea* (4.5–5.2 Mb). However, the SOL genome is larger than those of other Bacterioidetes symbionts, that is, the scale insect (Coccoidea: Hemiptera) symbiont *Ca.* Uzinura diaspidicola (28), the sap-feeding insect symbiont *Ca.* Karelsulcia muelleri (29) and the symbiont *Rhyzopertha dominica* (RdSym) (30, 31).

SOL has fewer amino acid biosynthetic pathways and relies on the host or its microbiome for essential nutrients. In return, SOL can supply the host with lipoic acids, pantothenate, and menaquinone via the futalosine pathway, similar to *S. canadensis* and *Solitalea lacus*, as indicated by KEGG database analysis (37). In this study, we were not able to distinguish whether these metabolites are provided to the host or are produced for SOL itself. However, the latter pathway was expressed at low levels. Compared with the genomes of other mite symbionts, the *Cardinium* (cTPut) genome encodes the lipoic acid pathway (14), whereas both the lipoic acid and pantothenate pathways are present in *Bartonella*-like symbionts (38).

### Interactions of *Candidatus* Krakonobacterium acarorum with *Cardinium* based on correlations of gene expression

According to previous findings, *Cardinium* modifies the gut microbiome of its host (39, 40), although it is located mainly in the reproductive tract. In this study, we investigated potential interactions between SOL and cTPut in *T. putrescentiae* by examining correlative gene expression data. In the cTPut-negative samples, the SOL read abundance was slightly higher than in the cTPut-positive samples, suggesting that cTPut may negatively influence SOL abundance. Overall, SOL gene expression did not significantly differ between samples with and without cTPut. However, statistically significant correlations were detected between gene expression in cTPut and that in SOL. For example, the expression of ABC membrane transporters in SOL was correlated with the expression profiles of several cTPut genes, mostly genes involved in genetic information processing. As ABC membrane transporters may be related to the transport of nutrients (such as lipids) and genetic information processing is likely related to growth, this suggests that SOL growth may be nutrient limited, with the presence of cTPut intensifying competition between the two bacteria for nutrients provided by the mite host. Recent studies have shown that the growth of intracellular symbionts is limited by carbohydrate intake (41), supporting nutrient competition between SOL and cTPut.

### Tripartite interactions among *Candidatus* Krakonobacterium acarorum, *Cardinium,* and their host *T. putrescentiae*

The effects of *Cardinium* on host immunity-related and regulatory pathways and metabolism have been previously reported (39, 42, 43). Furthermore, our correlation analyses provide evidence of distinct interactions between SOL and mite immunity-related and regulatory pathways in samples with and without cTPut. The mite peroxisome, autophagy, sphingolipid, apoptosis, PI3K–Akt, and lysozyme pathways exhibited much stronger correlations with SOL gene expression in the presence of cTPut than in the absence of cTPut (Table 3). The explanation for this is that, within the host, SOL symbionts are regulated by the mite phagocytosis and lysosome pathways in both the gut and other internal organs, as well as the fat body—typical locations where SOL is found (19). Some insect hosts utilize autophagy and apoptosis for symbiont regulation or recycling (44, 45). The presence of cTPut can stimulate such a host immune response. Additionally, our correlation analyses revealed that the presence of cTPut reduced the interaction between SOL and the mite host, as indicated by the decrease in the number of correlations between the host and SOL. This finding is consistent with previous models that described the impact of *Cardinium* on a different bacterium (*Wolbachia*) through the disruption of mite‒host interactions (14). Although the correlation data indicate that SOL and mite metabolism are strongly correlated, the presence of cTPut partly disturbs the interaction between SOL and mite metabolism. The differences in metabolic pathway expression were low in the presence/absence of cTPut.

In summary, our correlation-based gene expression analyses revealed (i) nutrient competition between SOL and cTPut and (ii) manipulation of SOL due to cTPut interacting with the mite host, resulting in changes in the host’s immunity-related/regulatory pathways, causing indirect effects on SOL.

## MATERIALS AND METHODS

### Samples of mites and feces

Cultures of *T. putrescentiae* and *A. siro* (Table 6) were maintained at the Czech Agrifood Research Center (Crop Research Institute until 2024), Prague, Czechia, and in a laboratory in China as described previously (46, 47). Mites were harvested from 1-month-old cultures maintained under controlled conditions at 25°C and 85% RH in darkness and then fed a wheat germ-derived diet (SPMd) (48). Adult mites were harvested with a brush from the surfaces and plugs of the flasks, and eggs and juveniles were found in the food at the bottoms of the rearing flasks. Mites were transferred into sterile tubes and weighed; samples with fresh weights ranging from 30 to 40 mg were used. Individual mite samples were processed following a previously published protocol (18, 49). SPGM was used as the source of feces. The SPGM is the fraction that contains diet debris, feces, and the remains of mite bodies after cultivation. The residual live mites and/or eggs were removed from the SPGM samples by sieving (50). The weights of the samples were equivalent to those of the live mites (30–40 mg). Eggs were collected following a protocol described previously (18). These samples were then used for genomic/transcriptomic sequencing and analyses, population-level qPCR with SOL-specific primers for mite and SPGM samples, and PCR with SOL-specific primers for single mites (Table 6). All the samples were surface sterilized on ice. Mites were cleaned by sequentially placing them in 100% ethanol, 0.5% sodium hypochlorite, and ddH_2_O following the protocol described previously (27).

**TABLE 6:** Cultures of the stored-product mites *Acarus siro* and *Tyrophagus putrescentiae* used in this study and the presence of intracellular symbionts^*a*^^,^^*b*^^,^^*c*^^,*d*^

ID	SP	Culture	Collector	Year	Diet	Site	IP	Genome	*Trans*	PCR	qPCR
	
5K	TP	Koppert	E. Baal	2012	SPMd	Koppert rearing facility, Netherlands	No	X	X	X	X
5L		Laboratory	E. Zdarkova	1996	SPMd	Grain, Bustehrad, Czechia	cTPut	X	X	X	X
5N		Dog	J. Hubert	2007	F	Food producing factory, St. Louis, Missouri, USA	wTPut	X	X	X	X
5P		Phillips	T. W. Phillips	2014	SPMd	Laboratory strain, Manhattan, Kansas, USA	wTPut	X	X	X	X
5Pi		Biscuit	M. Nesvorna	2015	SPMd	Biscuits contamination, Prague, Czechia	No	X	X	X	X
5S		Ham	A. Sala	2013	SPMd	Food-producing factory, Cesena, Italy	cTPut	X	X	X	X
5Tk		Teplice feed	M. Nesvorna	2015	SPMd	Horse feed contamination, Teplice, Czechia	No	X	X	X	X
CH		Laboratory	Z.-G. Liu	2017	SPMd	Laboratory culture, China	cTPut	X			
6L	AS	Laboratory	E. Zdarkova	1996	SPMd	Grain, Bustehrad, Czechia	No	X			X
6TK		Teplice feed	M. Nesvorna	2015	SPMd	Horse feed contamination, Teplice, Czechia	No				X
6Z		Zvoleneves	M. Nesvorna	2011	SPMd	Oil rapeseed debris, Zvoleneves, Czechia	No	X			
6Tu		Tuchomerice	M. Nesvorna	2016	SPMd	Rabbit feed contamination, Tuchomerice, Czechia	No				X

^*a*^ AS*—Acarus siro*, TP*—Tyrophagus putrescentiae*, F—dog kernel, IP—intracellular parasites, SP—species of mite, SPMd—diet of stored-product mites, cTPut*—Cardinium* symbiont of *T. putrescentiae*, wTPut*—Wolbachia* symbiont of *T. putrescetiae.*

^*b*^ The samples are described in detail in Table S15.

^*c*^ X indicates presence.

^*d*^ Empty cells indicate not observed.

### Prevalence of SOL in mite cultures

Two levels of quantification were performed: (i) single-mite-level quantification via conventional PCR (49) and (ii) population-level quantification via qPCR. Both reactions were conducted with SOL-specific primers. Conventional PCR (primers Soli_F and Soli_R) was used for single-mite analysis, with 30 individuals used as replicates per culture. This PCR generated an approximately 600 bp product (19). Each reaction contained 12.5 µL of EmeraldAmp MAX HS PCR 2× Master Mix (catalog number RR330A, TaKara, Kyoto, Japan), 8.5 µL of dH_2_O, 0.4 µM each primer and 2 µL of mite lysate, with a total reaction volume of 25 µL. Every PCR run contained a positive control (genomic DNA) and a negative control (ddH_2_O). Samples that produced a 600 bp band via gel electrophoresis were considered SOL positive. The negative samples were those that did not produce bands with the Soli_F and Soli_R primers but generated an approximately 1,500 bp band of bacterial 16S DNA with the universal UF and UR primers (51).

Amplification by qPCR was carried out in a StepOnePlus Real-Time PCR System (Life Technologies, Grand Island, NY, USA) in 96-well plates using GoTaq qPCR Master Mix (Promega). SYBR Green (Bio-Rad Laboratories, Veenendaal, the Netherlands) was used as a double-stranded DNA (dsDNA)-binding dye following a previously described protocol (18, 52, 53). The SOL-specific primers Soli_3Q and Soli_3QR (19) were used to amplify a 180 bp fragment of 16S rRNA. The next analyses were performed with cTPut-specific primers and a protocol described previously (19). All reactions were conducted in technical duplicates, with six biological replicates from each mite culture, mite body, SPGM, and egg sample. Microbial gene abundance was normalized to per-mite, per-egg, and per-gram SPGM values. Before analysis, gene abundance data were log(10)-transformed, and read counts below the detection limit were replaced with zeros.

### SOL genomic assemblies

All samples (Table 1; Table S1) were homogenized for 30 s in a glass tissue grinder (Kavalier glass, Prague, Czechia) in 500 µL of lysis buffer on ice (38). DNA was extracted from the homogenates after overnight incubation with 20 µL of proteinase K at 56°C using a QIAamp DNA Micro Kit (Qiagen, Hilden, Germany; cat. no. 56304) following the manufacturer’s protocol for the tissue samples. The samples were further processed in the Mr. DNA Laboratory (Shallowater, TX, USA). The concentrations of the extracted DNA samples were quantified using a Qubit dsDNA HS Assay Kit (Life Technologies), and the quality of the DNA was determined using a NanoDrop 2000 instrument. The samples were sheared in Covaris G-tubes (Covaris Inc.). The average size of the sheared DNA was determined using a TapeStation 4200 system (Agilent Technologies, Santa Clara, CA, USA). Paired-end libraries were prepared using a Nextera DNA Flex library preparation kit (Illumina) and sequenced for 500 cycles on a NovaSeq 6000 platform (Illumina). The long reads were obtained by PacBio sequencing. The libraries were prepared with the SMRTbell Express Template Prep Kit 2.0 (Pacific Biosciences) and sequenced on a PacBio Sequel system (Pacific Biosciences) (see Table S15).

Illumina DNA reads were trimmed using Trim Galore (54), and their quality was assessed with fastQC (55). The reads were *de novo* assembled using SPAdes v3.13.1 in metagenomic mode. Contigs were processed by PROKKA in metagenomic mode. PROKKA-predicted protein sequences were annotated using GhostKOALA, a tool that annotates protein functions by mapping sequences to the KEGG database (56). The obtained scores and taxonomic identification according to GhostKOALA were compared. Bacteroidota (=Bacteroidetes) contigs were selected, and Bacteroidetes contigs identified as *Cardinium* were discarded. Short reads were mapped using Bowtie2 (57, 58), and long reads were mapped using Minimap2 (59) against selected contigs. Filtered reads were *de novo* assembled using SPAdes in careful mode. The assembled genomes were polished using Pilon (60) and annotated using Prokka (61) on the DFAST webserver (62). KEGG proteins were identified using GhostKOALA and assigned to KEGG categories and metabolic pathways using KEGG Mapper (63). The genomes were visualized using Proksee (64) and VirSoter (65). The genomes were compared using the MASH algorithm (66) in dRep (67).

### Phylogenomic and sequence-based identification of SOL

We analyzed the whole genomes and 16S rDNA sequences of SOL (Table S4). The sequences were aligned using SINA online tools at Silva-arb (68), and a maximum likelihood phylogenetic tree was inferred using PHYML 3.0 (69) and 100 bootstrap replicates. Whole-genome taxonomic analyses were performed using the MASH algorithm (66) in the Type (Strain) Genome Server (TYGS) at https://tygs.dsmz.de (25, 26) integrated with the List of Prokaryotic names with Standing in Nomenclature (LPSN, https://lpsn.dsmz.de) (26). For whole-genome phylogenetic analyses, Bacteroidota (=Bacteroidetes) genomes were downloaded from GenBank (Table S4 and S5), and the analysis was performed using the MASH algorithm (66) in dRep (67). The next analysis was performed in M1CR0B1AL1Z3R (24) by detecting ORFs, finding orthologous groups, aligning orthologous sequences (70, 71), and inferring a maximum likelihood phylogenetic tree using RAxML with 100 bootstrap replicates (72). All trees were rooted and visualized in iTOLv 6 (73).

### Transcriptome samples and gene expression analyses

The mite meta-transcriptome samples included samples from *T. putrescentiae* cultures 5L, 5Pi, 5S, and 5Tk, with seven replicates per culture (Table S15), as described previously (16, 38). The homogenization procedure was the same as that used for the DNA samples. RNA extraction was performed with a NucleoSpin RNA Kit (catalog no. 740984.50; Macherey-Nagel, Duren, Germany) with the following modifications: homogenized samples were centrifuged at 2,000 × *g* for 3 s, and DNA was degraded with DNase I at 37°C according to the manufacturer’s protocol (Riboclear plus, catalog no. 313-50; GeneAll, Lisbon, Portugal). The RNA quality was evaluated using a NanoDrop instrument (NanoDrop One; Thermo Scientific, Waltham, MA, USA) and an Agilent 2100 Bioanalyzer (Agilent Technologies). The high-quality samples were sent to the Mr. DNA Laboratory. Poly-A selection and library preparation were performed using KAPA mRNA HyperPrep Kits (Roche), and paired-end sequencing was performed for 500 cycles using a NovaSeq 6000 system (Illumina). Transcriptomic analyses of cTPut, SOL, and mite-predicted KEGG gene expression were performed in CLC Workbench 22 (Qiagen, Venlo, the Netherlands). The workflow included read trimming and expression analyses of reference genomes, including the cTPut (JAUEML01), SOL (this study), and *T. putrescentiae* (JBBPFL01, 5,838 KEGG-predicted genes) genomes. The total number of reads mapped to each predicted gene was used as the output from the expression analysis. The previously annotated *T. putrescentiae* KEGG gene expression data are available at https://zenodo.org/records/15172873. The proportion of SOL/mite number of mapped reads was used as an indicator of expression. SOL gene expression was standardized to 3,000 reads, and cTPut gene expression was standardized to 5,000 reads.

### Statistical analysis

Gene expression analyses were performed in R using the vegan package (74). Mite gene expression was tested for those genes assigned to KEGG pathways (75) as described previously (14). The two variables, that is, SOL gene expression and cTPut gene expression, were derived from our previous data (14). Two types of correlation analyses were performed in PAST (76): dbRDA and Spearman correlation analysis.

The dbRDA was based on the comparison of two datasets describing gene expression, first as the dependent variable and second as the factor in robust Aitchinson distances. The ordistep function was used to select the factor variables (77). A Monte Carlo permutation test (10,000 permutations) was used to test the significance of the selected factors and models. The explained variability (*R*) of the models was used to assess the goodness of fit and determine the strength of the factors.

Spearman correlations were calculated for cTPut and SOL gene expression data from cTPut-positive samples (5S and 5L). We also performed Spearman correlation analyses for mite KEGG gene expression and SOL gene expression separately for cultures with cTPut (5S and 5L) and without cTPut (5Pi and 5Tk). These analyses were based on Spearman correlation with a permutation *P* value < 0.05. These Spearman correlation results were visualized using correlation maps generated with the Complex-Heatmap package (78, 79). These correlation data sets were also visualized in Cytoscape v.3.10 (80) with the Metscape plug-in (81). The numbers of positive and negative correlations for each analyzed gene were calculated and compared using a paired test in PAST.

## Data Availability

Our analyses are based on the following GenBank projects: PRJNA1051327, PRJNA1051327, PRJNA1074947, PRJNA1117590, PRJNA493156, PRJNA656450, PRJNA690683, PRJNA706095, and PRJNA990474. The SOL genomes are deposited under project PRJNA1196338.

## SUPPLEMENTAL MATERIAL

Supplemental figures spectrum.00609-25-s0001.docx Fig. S1 to S12.

Supplemental tables spectrum.00609-25-s0002.xlsx Tables S1 to S15.

ASM does not own the copyrights to Supplemental Material that may be linked to, or accessed through, an article. The authors have granted ASM a non-exclusive, world-wide license to publish the Supplemental Material files. Please contact the corresponding author directly for reuse.
