# Supplementary material for: Analysis of the tripartite interactions between two bacterial symbionts, a novel Solitalea-like bacterium (Bacteroidota) and Cardinium, and the stored product mite Tyrophagus putrescentiae based on gene expression data
Source: Microbiol Spectr. 2025 Jun 16;13(8):e00609-25. doi: 10.1128/spectrum.00609-25 (PMC12323627; doi:10.1128/spectrum.00609-25)
Supplement: Supplemental figures — Fig. S1 to S12. [file spectrum.00609-25-s0001.docx]

**Supplementary figures S1–S12**

**Analysis of the tripartite interactions between two bacterial symbionts, a novel *Solitalea*-like bacterium (Bacteroidota) and *Cardinium*, and the stored product mite *Tyrophagus* *putrescentiae* based on gene expression data**

**J. Hubert, Q. Xiong, E. Glowska-Patyniak, E. V. Furtak, P. B. Klimov**

**Microbiology Spectrum**

**FIG S1** Quantification of SOL in mite bodies or SPGM based on qPCR with SOL-specific primers: (**A**) Mite bodies, (**B**) SPGM: AS – Acarus siro and TP – Tyrophagus putrescentiae. The log-transformed read counts are shown as jitter boxplots. The number of copies was recalculated per mite or per gram of rearing medium. SPGM refers to debris from rearing medium, feces, and fragments of mite bodies after mite cultivation without living mites/eggs (which were removed by sieving). The numbers of SOL copies in the eggs were below the detection threshold of qPCR and are not shown.


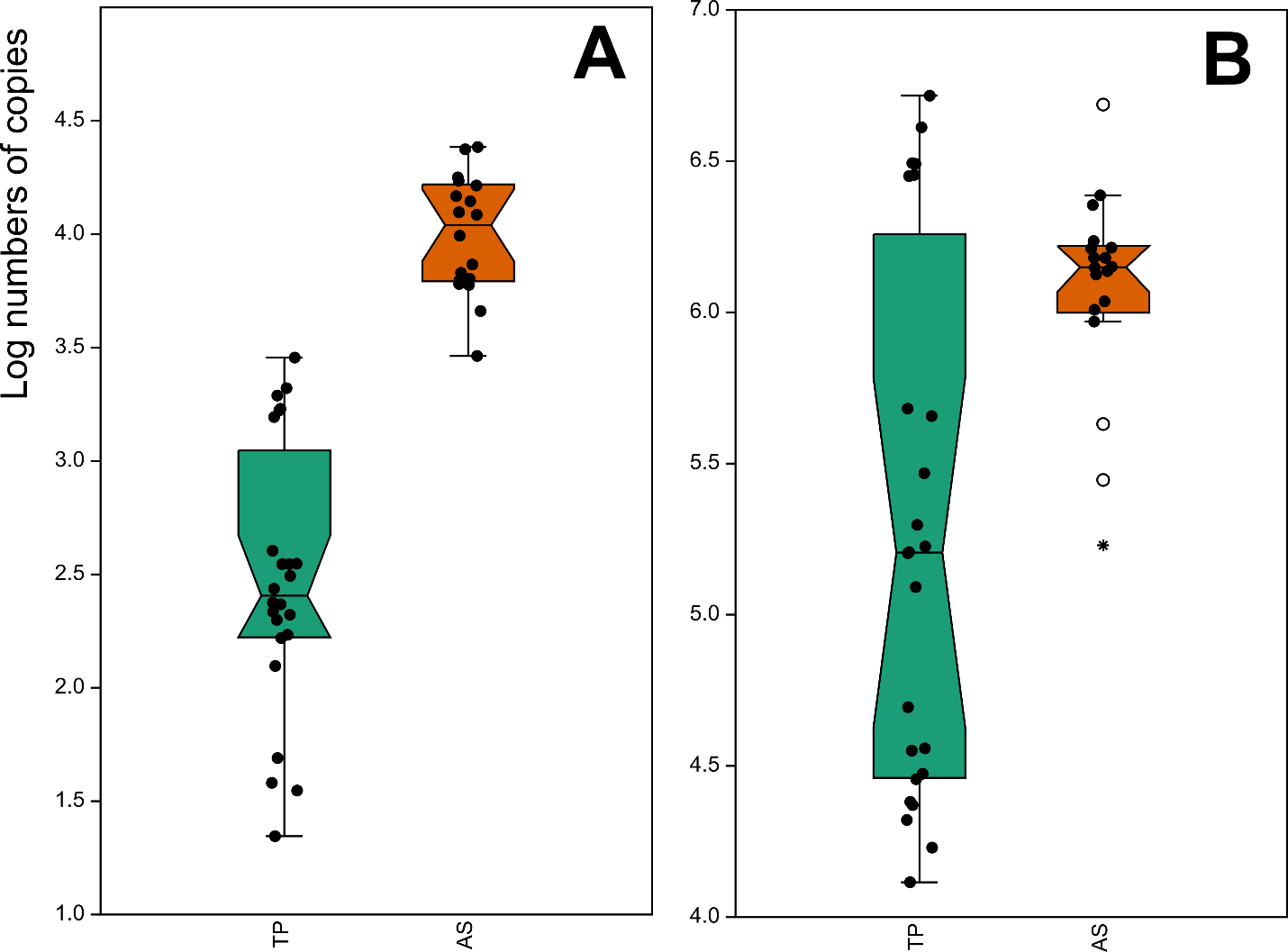


**FIG S2** Comparison of two assemblies of a new *Solitalea*-like symbiont (SOL) from *Tyrophagus putrescentiae* and *Acarus siro* using average nucleotide identity (ANI) (1); A – Czech vs. Chinese strains of *T. putrescentiae*; B – *T. putrescentiae* (Czechia) vs. *Acarus siro* strains.


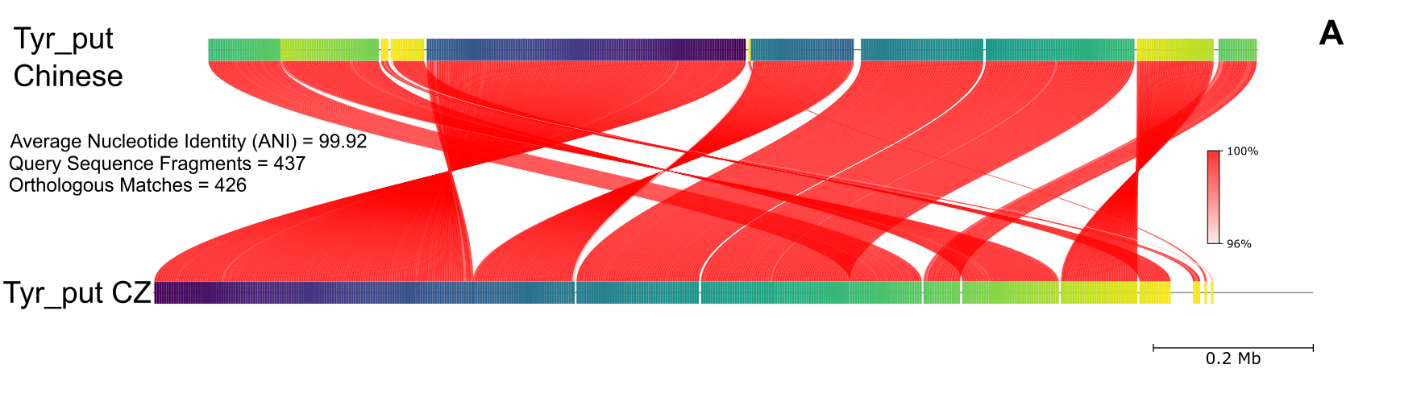

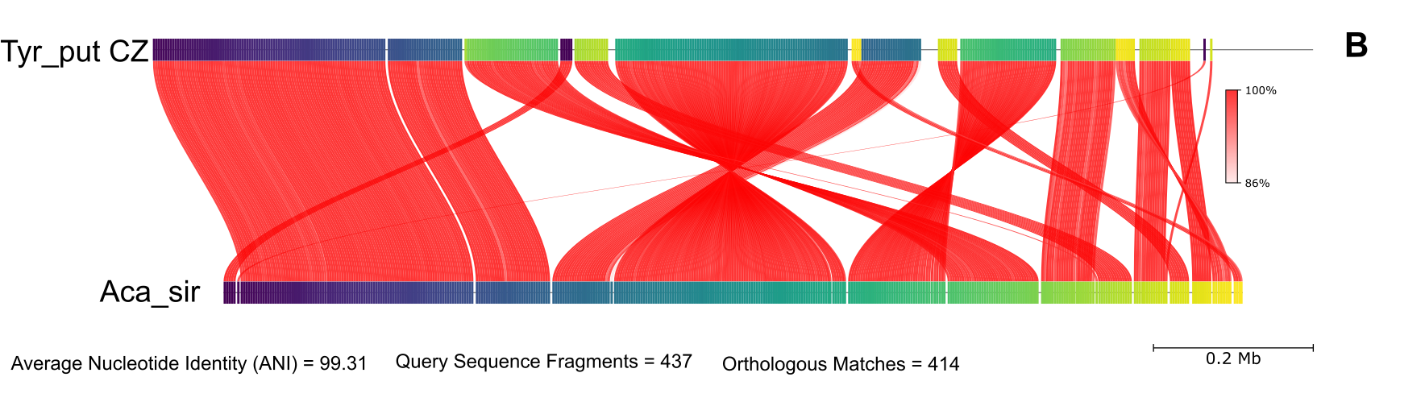


**FIG S3
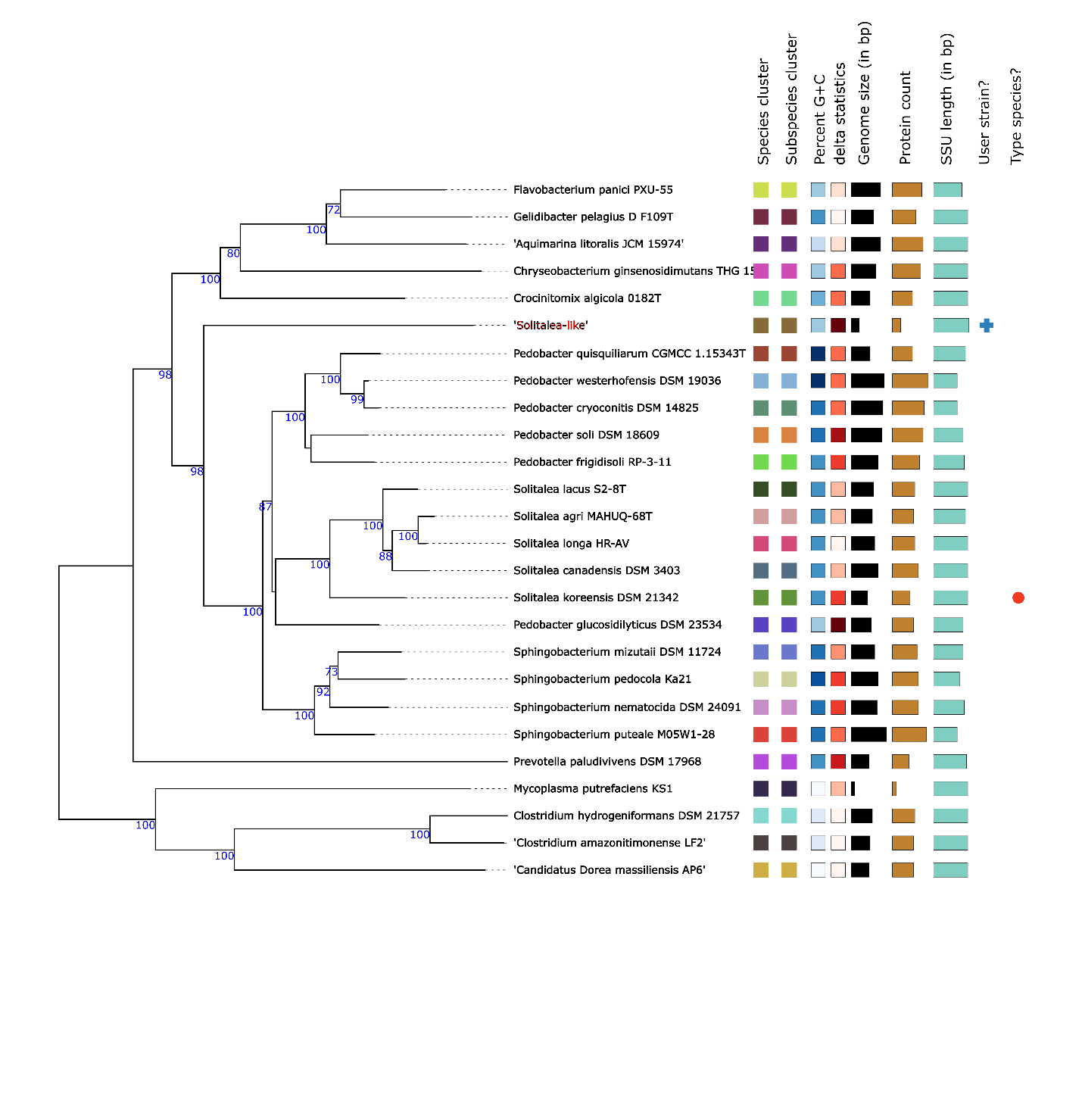
**Taxonomic identification of SOL (red color) based on the 16S rDNA gene using the Type Strain Genome Server (TYGS). The phylogenetic tree was inferred in FastME 2.1.6.1 (2) using GBDP (Genome BLAST Distance Phylogeny) distances. The branch lengths were scaled using GBDP distances (formula d5). The numbers above the branches are GBDP pseudobootstrap support values > 60% from 100 pseudobootstrap replicates. The tree was rooted at the midpoint (3).

**FIG S4** Taxonomic identification of SOL (red color) based on genomic data using the Type Strain Genome Server (TYGS). The phylogenomic tree was inferred in FastME 2.1.6.1 (2) from GBDP distances. The branch lengths were scaled using GBDP distances (formula d5). The numbers above the branches are GBDP pseudobootstrap support values >60% from 100 pseudobootstrap replicates. The tree was rooted at the midpoint (3).


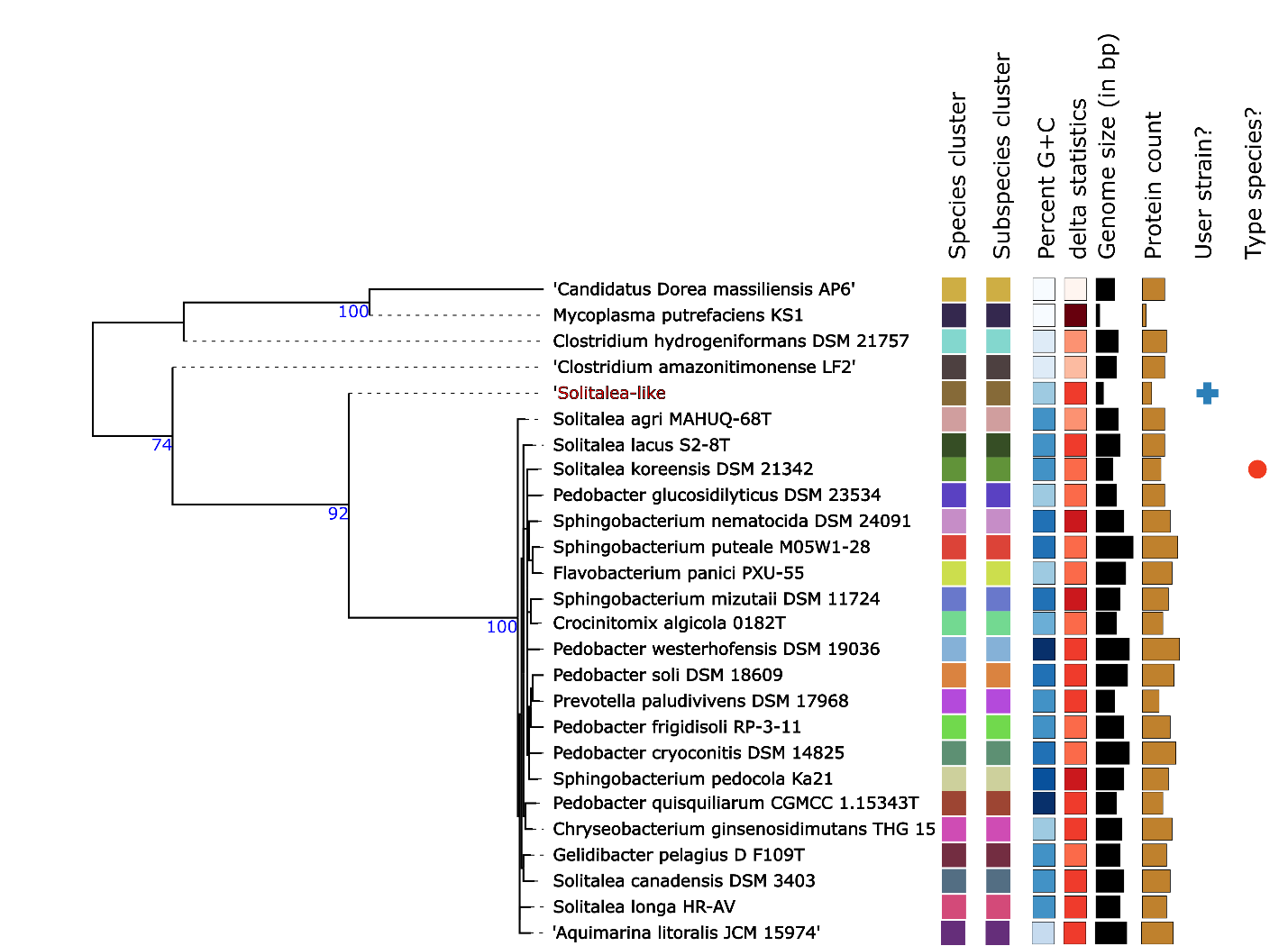


**
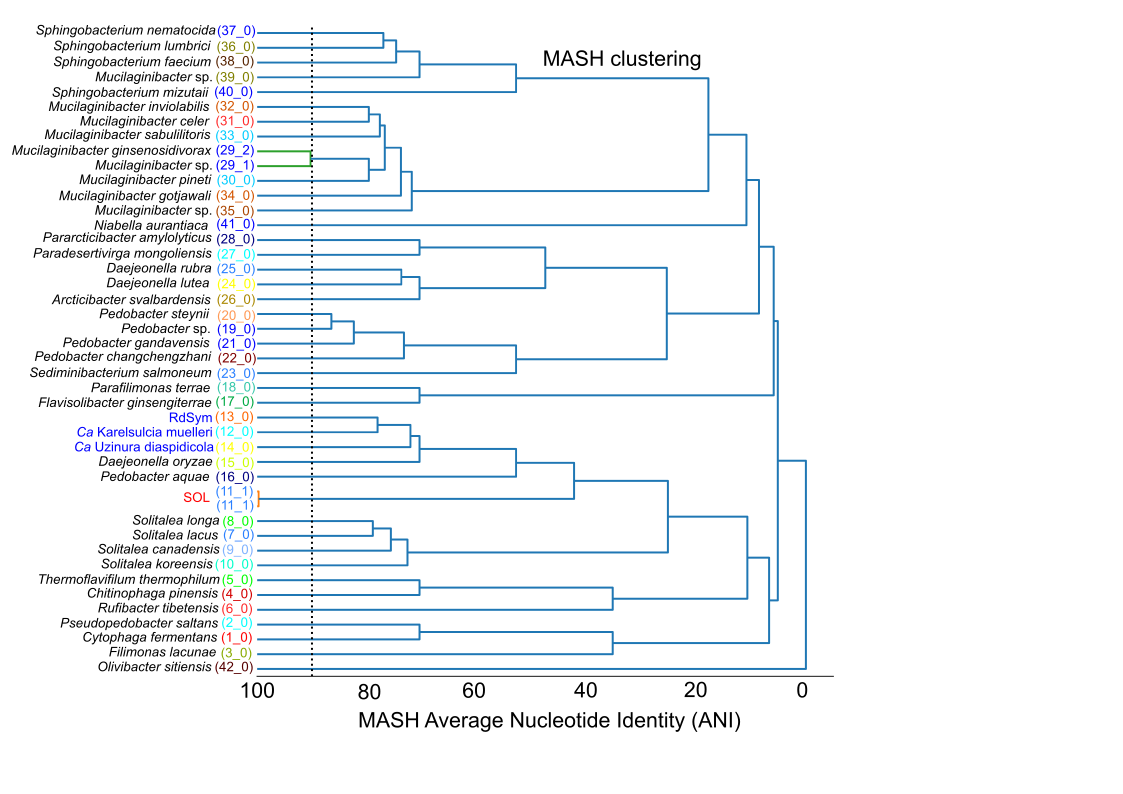
FIG S5** Phylogenomic tree based on MASH ANI-based clustering (Table S5: input data). The symbiotic bacteria are indicated in blue, and the SOL symbionts are indicated in red.

**
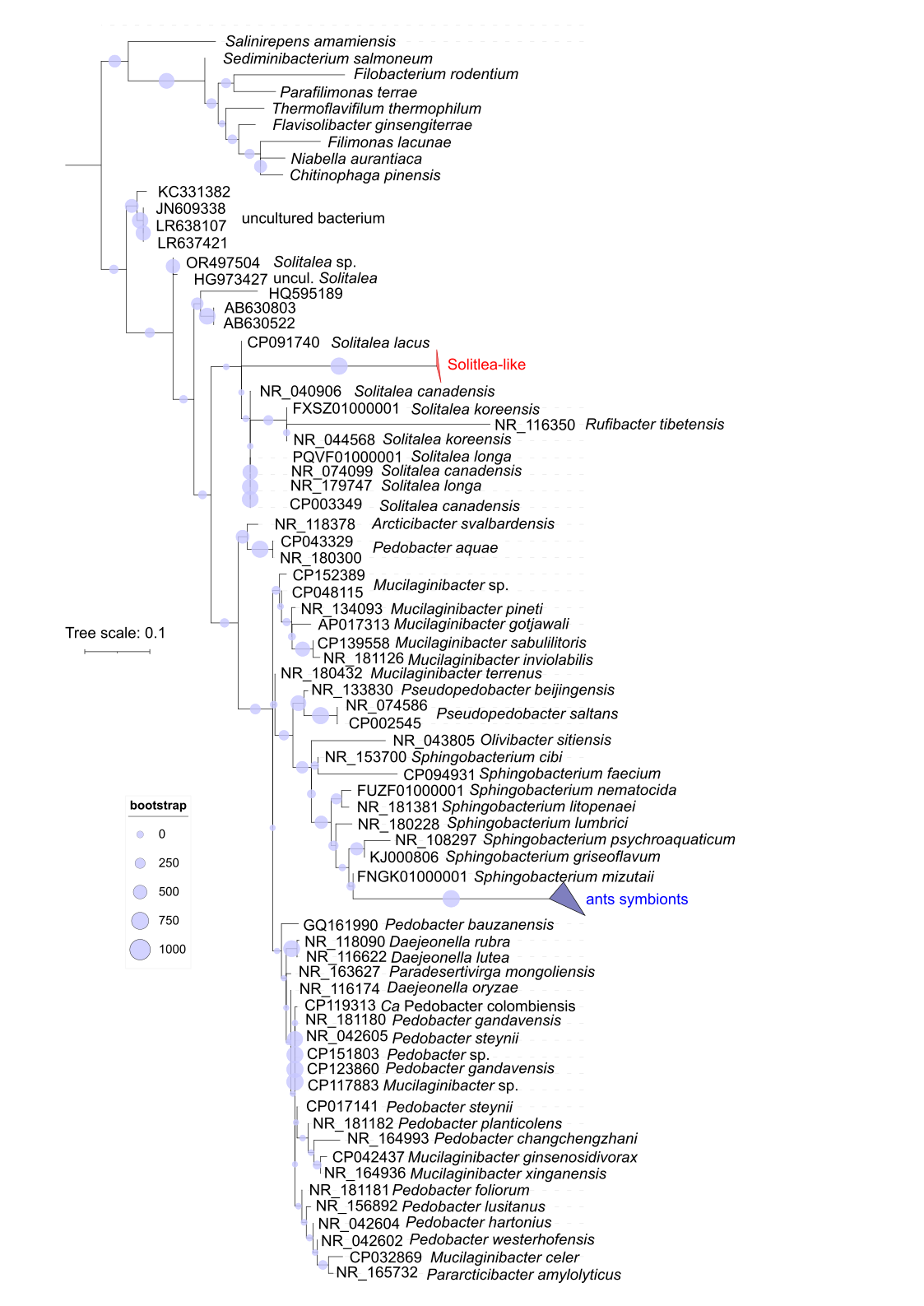
FIG S6** Identification of SOL based on a 253-bp 16S rDNA alignment. The phylogenetic tree was constructed using the GTR+R model with 1000 bootstrap replicates. The SOL symbiont, associated with mites, is highlighted in red, and ant symbionts are shown in blue (4, 5).

**FIG S7** Comparison of SOL gene expression in *Cardinium*-positive (cTPut) (5L and 5S) and *Cardinium*-negative samples (5Tk and 5Pi) from *Tyrophagus putrescentiae*; **A** – Relative abundance of SOL reads to mite reads; **B** – Shannon diversity index calculated from SOL gene expression. The data are visualized as box and jitter plots.

**
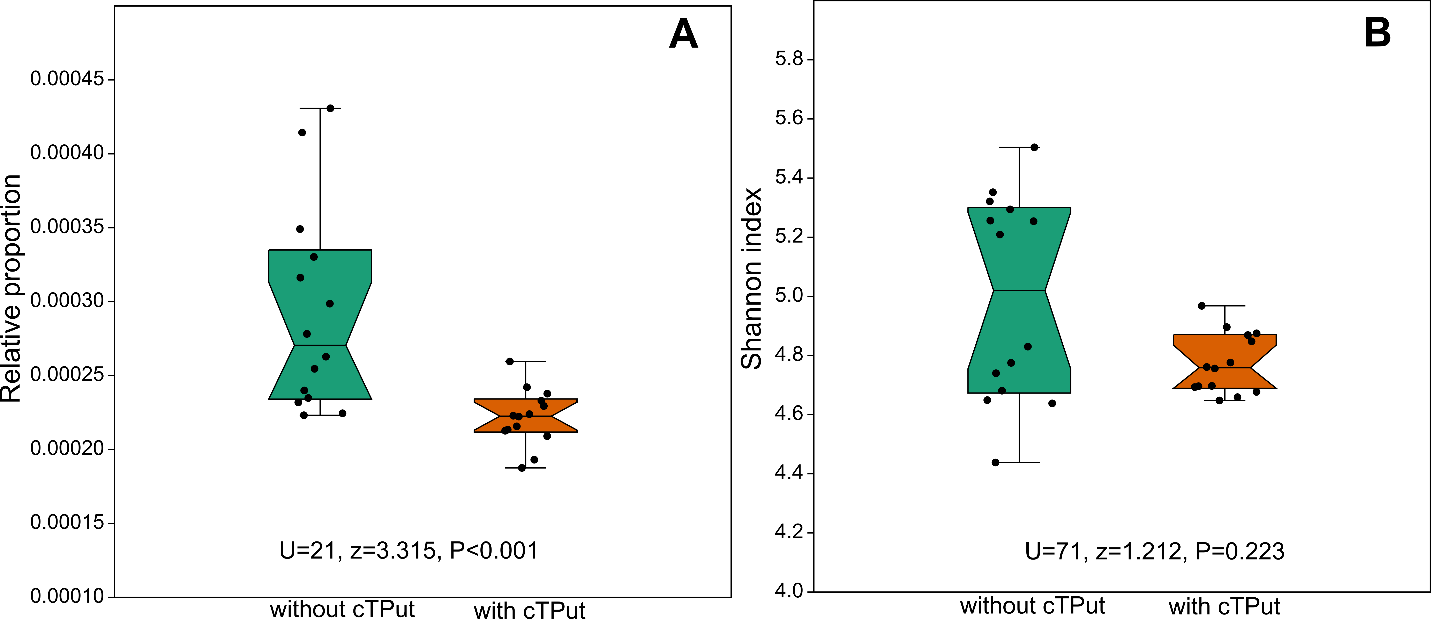
**


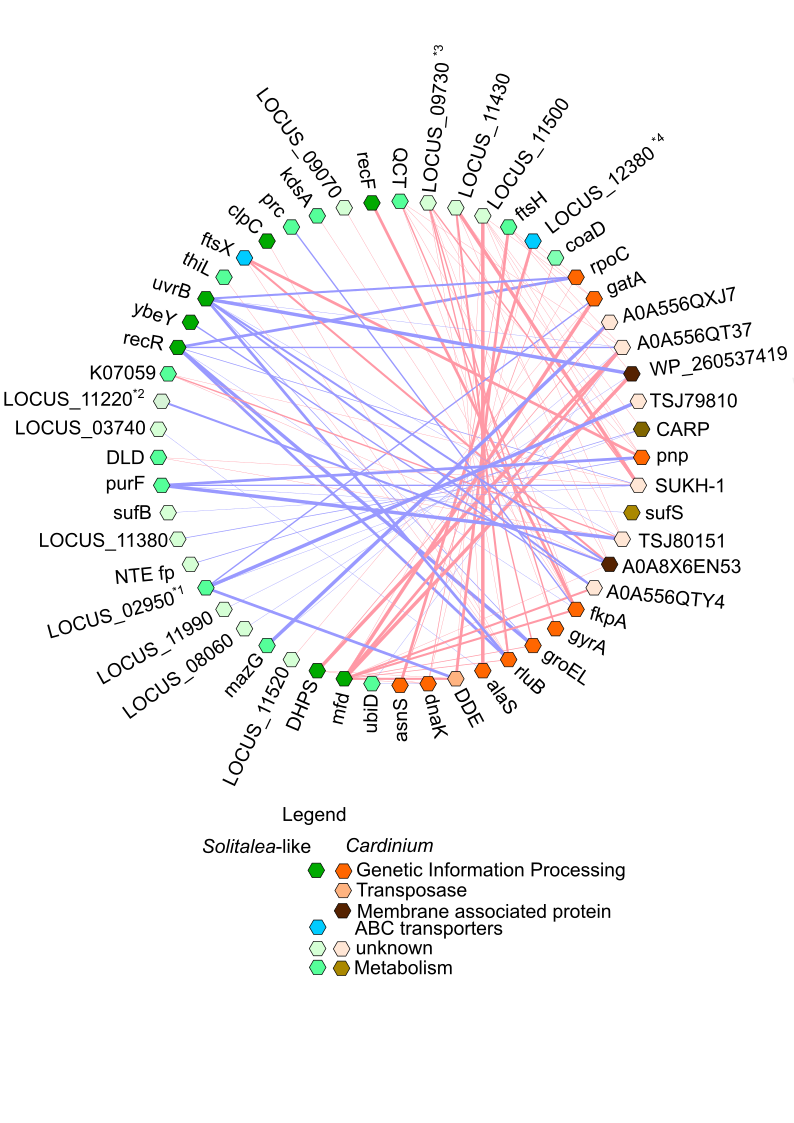
**FIG S8** Cytoscape gene expression correlation network between cTPut and SOL. The network includes Spearman correlations (permutation P<0.05) from the expression of SOL and cTPut, along with selected cTPut genes based on a high number of correlations with SOL. The network was constructed using Spearman correlation coefficients with absolute values of 0.75–1. Red indicates positive correlations, and blue indicates negative correlations. Line thickness represents the strength of the correlation.

**Notes:** ^*1^ glycosyltransferase family 9; ^*2^ TraG family conjugative transposon ATPases; ^*3^ Type II toxin-antitoxin system antitoxin, RelB/DinJ family; ^*4^ ABC transmembrane type-1 domain-containing proteins.

**FIG S9** Correlation heatmap based on Spearman correlation (permutational P<0.05) of SOL gene expression and KEGG gene expression data of the mite *Tyrophagus putrescentiae* in cTPut-negative cultures (5Pi and 5Tk).


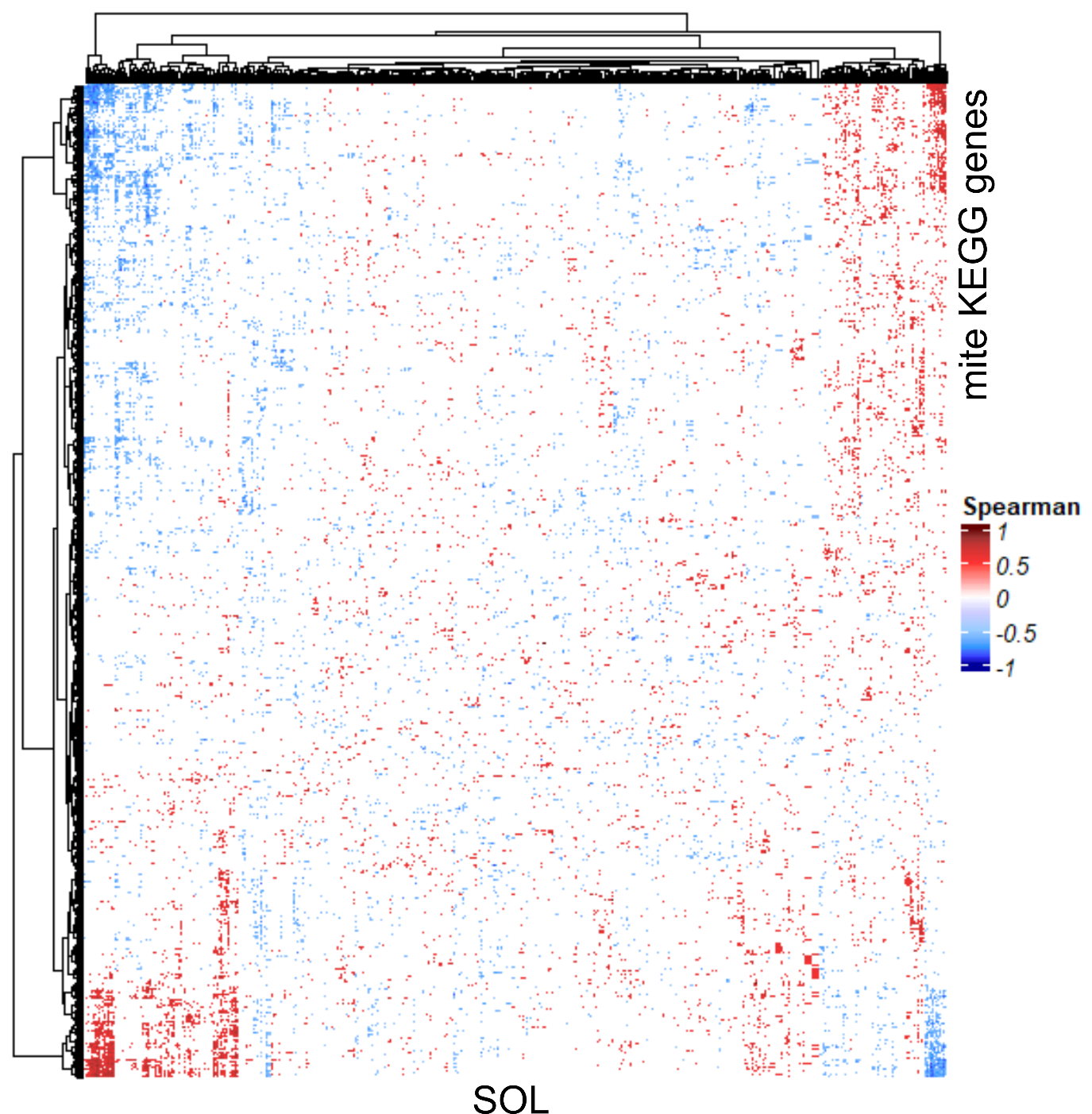


**FIG S10** Correlation heatmap based on Spearman correlations (permutational P<0.05) of SOL gene expression and KEGG gene expression data of the mite *Tyrophagus putrescentiae* in cTPut-positive cultures (5S and 5L).


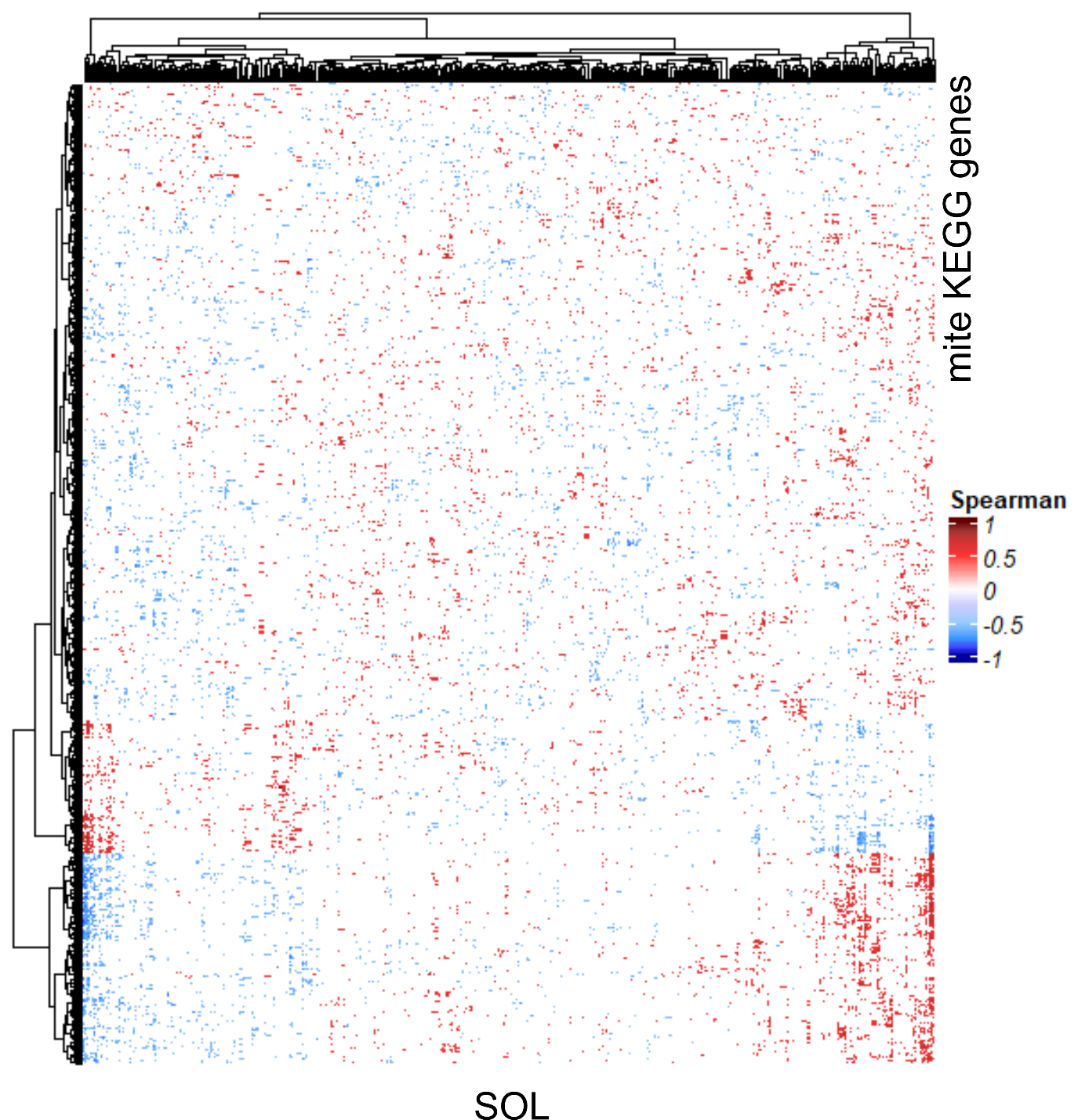


**
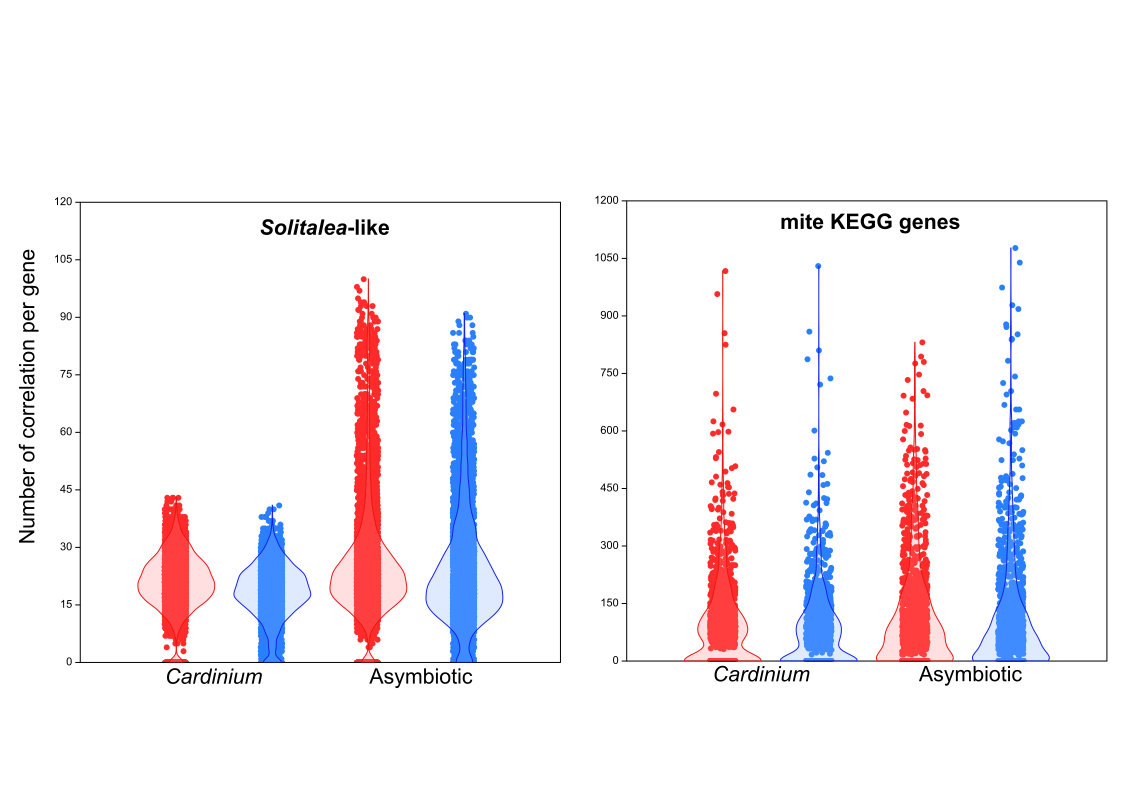
FIG S11** Comparison of the numbers of correlations per gene between SOL and *Tyrophagus putrescentiae-*predicted KEGG genes in cultures with and without cTPut (asymbiotic). The data are presented as violin and jitter plots. Red indicates a positive correlation, and blue indicates a negative correlation.

**
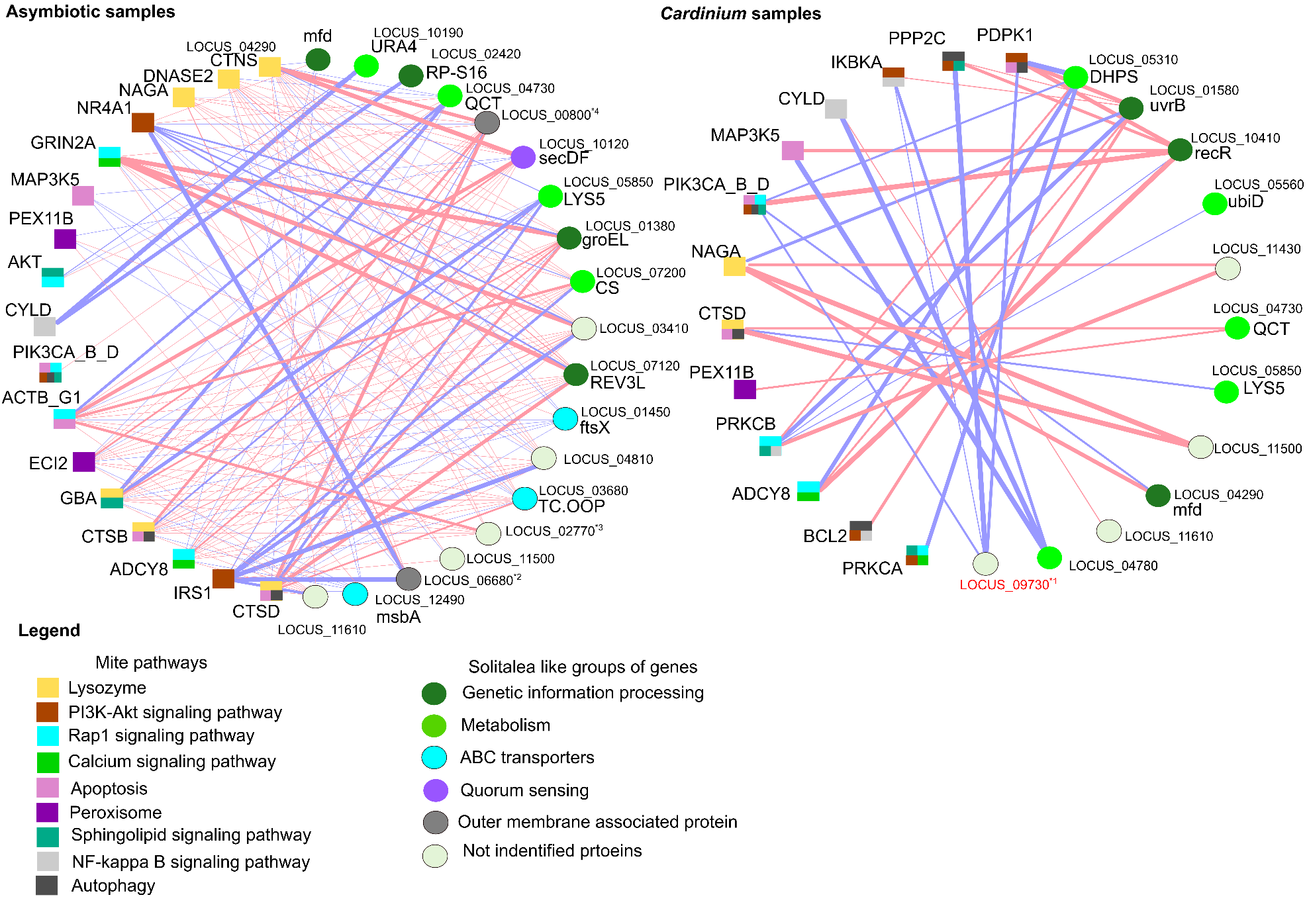
FIG S12** Correlation network between SOL and predicted mite KEGG genes in immune and regulatory pathways created in Cytoscape. The networks include Spearman correlations (permutational P < 0.05) from selected genes (see Table S9, total strength > 100) in samples with and without cTPut. The networks were constructed using Spearman correlation coefficients with absolute values of 0.75–1. Red indicates positive correlations, whereas blue indicates negative correlations. Line thickness represents the strength of the correlation. The data used to construct these networks are given in Table S11. Mite genes are shown as squares, and SOL genes are shown as circles.

**Notes:** ^*1^Type II toxin-antitoxin system antitoxin, RelB/DinJ family; ^*2^ Outer membrane protein beta-barrel domain-containing protein; ^*3^ HTTM-like domain-containing protein; ^*4^ Outer membrane protein OmpA.

**Supplementary references**

1. Jain C, Rodriguez-R LM, Phillippy AM, Konstantinidis KT, Aluru S. 2018. High throughput ANI analysis of 90K prokaryotic genomes reveals clear species boundaries. Nat Commun 9(1):5114. <https://doi.org/10.1038/s41467-018-07641-9>
2. Lefort V, Desper R, Gascuel O. 2015. FastME 2.0: a comprehensive, accurate, and fast distance-based phylogeny inference program. Mol Biol Evol 32(10):2798–2800. <https://doi.org/10.1093/molbev/msv150>
3. Farris JS. 1972. Estimating phylogenetic trees from distance matrices. Am Nat 106(951):645–668. <https://doi.org/10.1086/282802>
4. Cabuslay C, Wertz JT, Bechade B, Hu Y, Braganza S, Freeman D, Pradhan S, Mukhanova M, Powell S, Moreau C, Russell JA. 2024. Domestication and evolutionary histories of specialized gut symbionts across cephalotine ants. Mol Ecol 33(15):e17454. <https://doi.org/10.1111/mec.17454>
5. Hu Y, D'Amelio CL, Bechade B, Cabuslay CS, Lukasik P, Sanders JG, Price S, Fanwick E, Powell S, Moreau CS, Russell JA. 2023. Partner fidelity and environmental filtering preserve stage-specific turtle ant gut symbioses for over 40 million years. Ecol Monogr 93(1):e1560. <https://doi.org/10.1002/ecm.1560>
